# Strongly bound excitons in anatase TiO_2_ single crystals and nanoparticles

**DOI:** 10.1038/s41467-017-00016-6

**Published:** 2017-04-13

**Authors:** E. Baldini, L. Chiodo, A. Dominguez, M. Palummo, S. Moser, M. Yazdi-Rizi, G. Auböck, B.P.P. Mallett, H. Berger, A. Magrez, C. Bernhard, M. Grioni, A. Rubio, M. Chergui

**Affiliations:** 1 Laboratory of Ultrafast Spectroscopy, ISIC and Lausanne Centre for Ultrafast Science (LACUS), École Polytechnique Fédérale de Lausanne (EPFL), Lausanne, CH-1015 Switzerland; 2 Unit of Nonlinear Physics and Mathematical Modelling, Department of Engineering, Università Campus Bio-Medico di Roma, Via Álvaro del Portillo 21, Rome, I-00128 Italy; 3 Center for Life Nano Science @ Sapienza, Istituto Italiano di Tecnologia, Viale Regina Elena 291, Rome, I-00161 Italy; 4 Max Planck Institute for the Structure and Dynamics of Matter, Hamburg, D-22761 Germany; 5 Dipartimento di Fisica and INFN, Università Tor Vergata, Via della Ricerca Scientifica 1, Rome, I-00133 Italy; 6 Laboratory of Electron Spectroscopy, IPHYS and Lausanne Centre for Ultrafast Science (LACUS), École Polytechnique Fédérale de Lausanne (EPFL), Lausanne, CH-1015 Switzerland; 7 Department of Physics, University of Fribourg, Chemin du Musée 3, Fribourg, CH-1700 Switzerland; 8 Crystal Growth Facility, École Polytechnique Fédérale de Lausanne (EPFL), Lausanne, CH-1015 Switzerland; 9 Departamento Fisica de Materiales, Universidad del País Vasco, Av. Tolosa 72, San Sebastian, E-20018 Spain

## Abstract

Anatase TiO_2_ is among the most studied materials for light-energy conversion applications, but the nature of its fundamental charge excitations is still unknown. Yet it is crucial to establish whether light absorption creates uncorrelated electron–hole pairs or bound excitons and, in the latter case, to determine their character. Here, by combining steady-state angle-resolved photoemission spectroscopy and spectroscopic ellipsometry with state-of-the-art ab initio calculations, we demonstrate that the direct optical gap of single crystals is dominated by a strongly bound exciton rising over the continuum of indirect interband transitions. This exciton possesses an intermediate character between the Wannier–Mott and Frenkel regimes and displays a peculiar two-dimensional wavefunction in the three-dimensional lattice. The nature of the higher-energy excitations is also identified. The universal validity of our results is confirmed up to room temperature by observing the same elementary excitations in defect-rich samples (doped single crystals and nanoparticles) via ultrafast two-dimensional deep-ultraviolet spectroscopy.

## Abstract (web summary)

Here the authors combine steady-state angle-resolved photoemission spectroscopy, ellipsometry and ultrafast two-dimensional ultraviolet spectroscopy to examine the role of many-body correlations in anatase TiO_2_, revealing the existence of strongly bound excitons in single crystals and nanoparticles.

## Introduction

The field of excitonics has gained increased attention in the last years, due to the unique properties that excitons manifest in the conversion and transport of energy. Key to these developments is the ability to exploit exciton physics in materials that are easily fabricated and widely available. Anatase TiO_2_ belongs to a class of solids with superior functionalities for the conversion of light into other forms of energy^1–3^, but despite the wide effort dedicated to improve its optoelectronic performances, the microscopic nature of the fundamental electronic and optical excitations is still not understood. It is therefore pivotal to clarify the single-particle and two-particle excitation spectra of pure anatase TiO_2_, and to establish the nature of the charge excitations produced upon photon absorption.

**Fig. 1:**
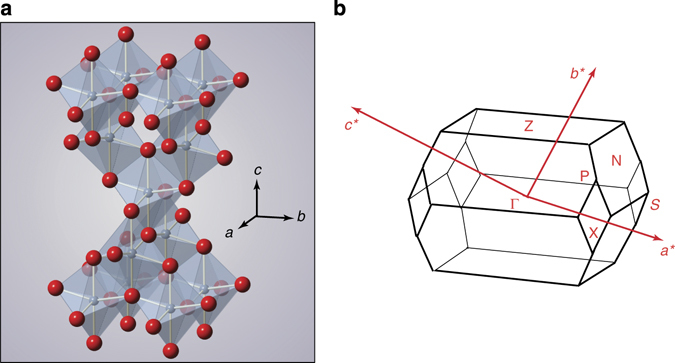
Anatase TiO_2_ crystal structure and BZ. **a** Crystallographic structure of anatase TiO_2_ with highlighted TiO_6_ polyhedra. *Blue atoms* represent titanium, *red atoms* represent oxygen. **b** Representation of the 3D BZ of anatase TiO_2_

Two key aspects of anatase TiO_2_ are: (i) it crystallises in a tetragonal unit cell, built on a network of corner-sharing or edge-sharing TiO_6_ octahedra (Fig. 1a), with a substantial difference between the lattice constants *a* = 3.78 Å and *c* = 9.51 Å; (ii) the Ti-3*d* O-2*p* orbital interactions run mainly in TiO_2_ bilayers perpendicular to the [001] direction, and display only a minor contribution along the *c*-axis^4^. This leads to an electronic structure with almost flat bands along the Γ-Z direction of the three-dimensional (3D) Brillouin zone (BZ) (Fig. 1b), and to a strong optical anisotropy for light polarised in the (001) plane and perpendicular to it.

First significant steps towards understanding the electronic excited states of this material were achieved by experimental probes such as angle-resolved photoemission spectroscopy (ARPES)^5, 6^ and optical spectroscopy^7–9^. Recent ARPES studies revealed that this material has an indirect bandgap, since the valence band (VB) maximum lies close to the X point and the conduction band (CB) minimum is at the Γ point of the BZ^5, 6^; consequently, the lowest optical absorption edge can be described in terms of an Urbach tail caused by the phonon-induced localisation of excitons^7^. Less experimental attention, however, has been paid to the detailed characterisation of the optical response above the absorption threshold, where anisotropy effects become more pronounced^8, 9^. In particular, the role played by many-body correlations in the optical properties has remained elusive to experimental probes, leading to a lack of knowledge about the nature of the elementary direct charge excitations in this material.

Many-body correlations have been investigated within the theoretical framework of density functional theory (DFT) with perturbation-theory corrections at the G_0_W_0_ level. This ab initio method provided a preliminary description of the material’s dielectric function^10–13^, despite neglecting the roles of doping, electron–phonon coupling, temperature effects and indirect transitions. The diagonalization of the Bethe–Salpeter Hamiltonian predicted several direct optical transitions at energies well below the direct electronic gap computed at the GW level. The existence of these bound localised excitons in anatase TiO_2_ is, however, still awaiting experimental verification, due to the difficulty of measuring the exciton binding energy (*E*_B_) for an indirect gap material. Indeed, conventional experimental techniques like optical absorption^14^, photoluminescence^15, 16^ and magneto-optics^17^ are not suitable, as the onset of the direct band-to-band transitions cannot be identified; moreover, to derive *E*_B_, these methods often rely on approximate models valid only for Wannier–Mott excitons^18^.

The present work uses a unique combination of steady-state and ultrafast experimental tools with advanced theoretical calculations, to unambiguously reveal the role of many-body correlations in anatase TiO_2_ and identify the nature of its elementary electronic excitations. By ARPES and spectroscopic ellipsometry (SE), we provide accurate values of the direct gap for both charged and neutral excitations. This leads us to unravel the existence of strongly bound excitons in this material and to offer a rigorous estimate of *E*_B_ for a direct exciton rising over the continuum of indirect (phonon-mediated) interband transitions, free from assumptions on the nature of the excitonic species under study. These results are supported by many-body perturbation-theory calculations, which include for the first time the role of doping, electron–phonon coupling and indirect transitions in this material. Our calculations confirm the stability of bound excitons and provide a complete description of their real-space behaviour. The room temperature (RT) robustness and generality of these elementary excitations is finally demonstrated by an ultrafast deep-ultraviolet (UV) two-dimensional (2D) spectroscopic study of widely different samples, ranging from single crystals with various degrees of doping to colloidal nanoparticles (NPs).

## Results

### Angle-resolved photoemission spectroscopy (ARPES)

**Fig. 2:**
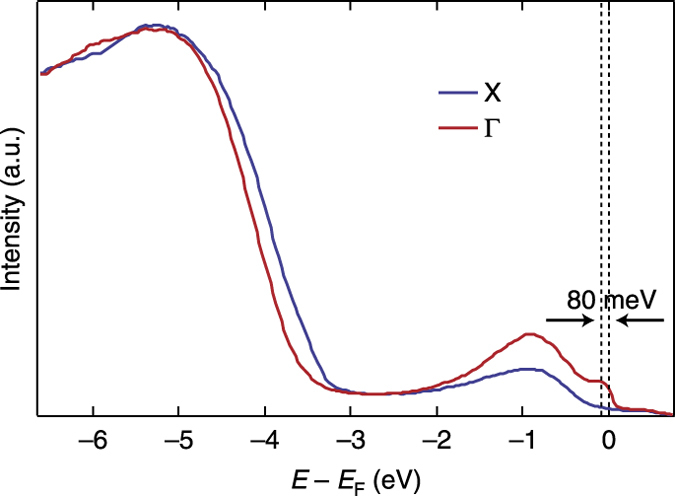
ARPES energy distribution curves. Energy distribution curves at the X (*blue curve*) and Γ (*red curve*) points of the BZ for a crystal at 20 K doped with an excess electron density *n* = 2 × 10^19^ cm^−3^. The spectra exhibit a feature at −1 eV, corresponding to the in-gap oxygen defect states. The curve at Γ exhibits spectral weight at *E*_F_, which lies 80 meV above the conduction quasiparticle band. The spectrum is referenced to *E*_F_

To reveal the possible existence of a direct exciton, an accurate determination of the direct electronic bandgap is needed. To this aim, we perform ARPES measurements on anatase TiO_2_ single crystals at 20 K, using a photon energy *hv* = 128 eV and a resolution of 30 meV. In particular, we introduce an excess electron density (*n*) in the CB of the material by inducing oxygen vacancies in a (001)-oriented single crystal, as described in ref. 6. As a consequence, the Fermi level (*E*_F_) pins slightly above the CB edge, providing a robust reference to measure the quasiparticle gap at the Γ point. To evaluate the shift of *E*_F_ above the CB edge, in Fig. 2 we show the energy distribution curves at the X (*blue line*) and Γ (*red line*) points of the 3D BZ for a crystal doped with an excess electron density *n* = 2 × 10^19^ cm^−3^. In both spectra, the structure arising at −1 eV corresponds to the well-established in-gap oxygen defect states^19^. The curve at Γ exhibits spectral weight at *E*_F_, which lies 80 meV above the conduction quasiparticle band.

**Fig. 3:**
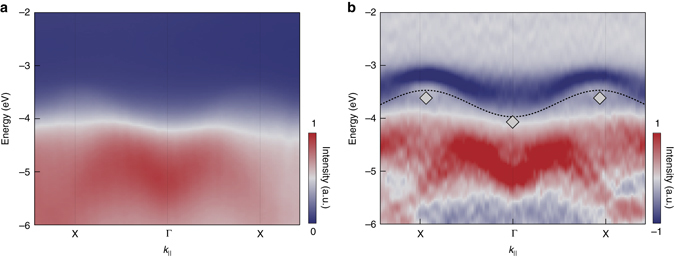
ARPES data of a single crystal of anatase TiO_2_. **a** ARPES energy vs. momentum intensity map for a crystal doped with an excess electron density *n* = 2 × 10^19^ cm^−3^ at 20 K. **b** Second derivative ARPES data of the electronic structure at the top of the VB between Γ and X. *Dashed blue lines* are added as a guide to the eye. The spectrum is referenced to the *bottom* of the CB at Γ. The intensity is indicated by a linear colour scale, as displayed in the colour bar

The valence states at 20 K along the Γ-X line are displayed in Fig. 3a and referenced to the bottom of the CB. To resolve the dispersive features present in the ARPES maps and evaluate the energy of the quasiparticle in the VB, we use the approach based on computing the second derivative of the ARPES data with respect to the energy (Fig. 3b)^20^. In this *panel*, the *dashed blue line*, highlighting the top of the VB, has been drawn as a guide to the eye. As expected, the VB onset occurs close to the X point, and precedes the rise at Γ by ~0.5 eV. Direct inspection of the band structure yields a first highly dispersing band close to the VB upper edges, whose maxima in the vicinity of the X and Γ points are at −3.47 ± 0.03 eV and −3.97 ± 0.03 eV, respectively, representing valuable estimates of the quasiparticle energies.

We also focus on the Γ point and monitor the evolution of the quasiparticle gap as a function of doping, by performing ARPES measurements at variable excess electron density (5 × 10^17^ cm^−3^ ≤*n* ≤ 5 × 10^20^ cm^−3^) in the CB. The energy–momentum dispersion relations for the bottom of the CB in the two extreme *n*-doping levels considered in our experiment are shown in Supplementary Fig. 3a,b; the second derivatives of these maps with respect to the energy axis are shown in Supplementary Fig. 3c,d. The latter allow us to identify the position of the quasiparticle energy at the bottom of the CB. Supplementary Fig. 3e compares the energy distribution curves at the Γ point of the BZ at the two considered doping levels. Relying on the second derivative analysis also for the VB, we can estimate the energy difference from the quasiparticle VB maximum to the CB minimum at Γ for the two doping levels at ~3.98 eV. This shows that there is no measurable bandgap renormalization (BGR) at the Γ point upon increased electron concentration over three orders of magnitude. This observation is also supported by many-body perturbation theory results (*see* below).

### Spectroscopic ellipsometry (SE)

**Fig. 4:**
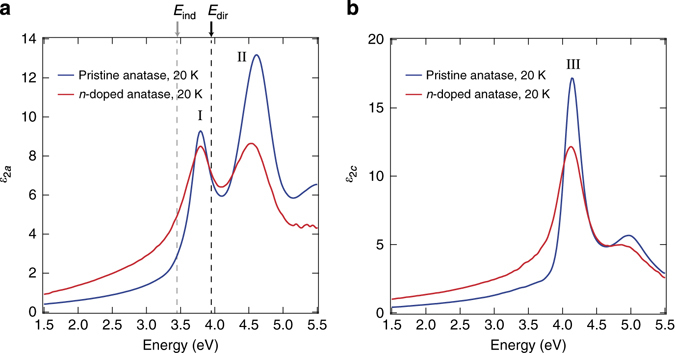
Optical spectra of anatase TiO_2_ single crystals. Imaginary part of the dielectric function at 20 K with the electric field polarised along **a** the *a*-axis (**E** ⊥ c) and **b** the *c*-axis (**E** ǁ c). The experimental data measured by SE on a pristine (*n *~ 0 cm^−3^) anatase TiO_2_ single crystal are reported in *blue*, while those obtained on a highly n-doped single crystal (*n* = 2 × 10^19^ cm^−3^) in *red*. The quasiparticle indirect gap *E*_ind_ = 3.47 eV and direct gap *E*_dir_ = 3.97 eV, as estimated by ARPES, are indicated by *dashed grey* and *black vertical lines*, respectively

As far as the direct gap of the two-particle excitation spectrum (i.e., the optical spectrum) is concerned, a very reliable experimental technique for measuring the dielectric function *ε*(*ω*) = *ε*_1_(*ω*) + i*ε*_2_(*ω*) of a material is SE. Figure 4a,b shows the imaginary part of the dielectric function, *ε*_2_(*ω*), measured at 20 K on (010)-oriented single crystals, with light polarised perpendicular (**E** ⊥ c) and parallel (**E** ǁ c) to the *c*-axis, respectively. The spectra are obtained both on a pristine (*n*~0 cm^−3^) crystal (*blue lines*) and on the same n-doped crystal used for the ARPES measurement (*n* = 2 × 10^19^ cm^−3^) (*red lines*). In the pristine crystal, the direct absorption for **E** ⊥ c (Fig. 4a) is characterised by the presence of a sharp peak at 3.79 eV (I), preceded by a long Urbach tail at lower energies^7^. A second, broader charge excitation (II) lies at 4.61 eV and extends up to 5.00 eV. The *c*-axis response (Fig. 4b) is instead characterised by a feature peaking at 4.13 eV (III) with a shoulder at 5.00 eV. Remarkably, all these excitations are still clear-cut in the n-doped sample, where we observe: (i) no apparent shift in the peak energy of features I and III, and an 80 meV redshift in the peak energy of feature II; (ii) a reduction of the oscillator strength of all peaks, due to a transfer of spectral weight from the above-gap to the below-gap region; (iii) a pronounced broadening of the spectral features.

From Fig. 4, we conclude that the lowest direct optical excitation, i.e., the direct optical gap of the material, is the feature at 3.79 eV for both pristine and n-doped (*n* = 2 × 10^19^ cm^−3^) anatase TiO_2_. Combined with the ARPES data, we also conclude that it belongs to a bound exciton of the n-doped single crystal, with an *E*_B_ = 180 meV, which is experimentally derived for the first time. We also investigate the temperature effect on the stability and renormalization of exciton I, and compare the dielectric function of pristine anatase TiO_2_ at 20 and 300 K (Supplementary Fig. 6). Peak I is found to undergo a blueshift of 40 meV from 20 to 300 K, while peak II undergoes a small redshift. The detailed interpretation of the full temperature dependence will be a subject of a separate publication; but here, we briefly stress that this observation implies the stability of exciton I even at RT. In addition, the blueshift of this peak is anomalous^21^, because it is opposite to the trends observed in standard insulators^22^. Blueshifts of high-energy electronic excitations have previously been reported in only few materials, and explained phenomenologically by invoking different aspects related to the electron-phonon coupling: The temperature dependence of the Debye–Waller factors in PbTe^23^, the *p*-*d* hybridisation modulated by the electron–phonon interaction in chalcopyrites^24, 25^, the Fröhlich interaction in perovskite titanates^26^. We will show below that, by taking into account the role of the electron–phonon coupling and temperature effects, our ab initio calculations are able to reproduce the anomalous blueshift of exciton peak I.

### Many-body perturbation theory calculations

**Fig. 5:**
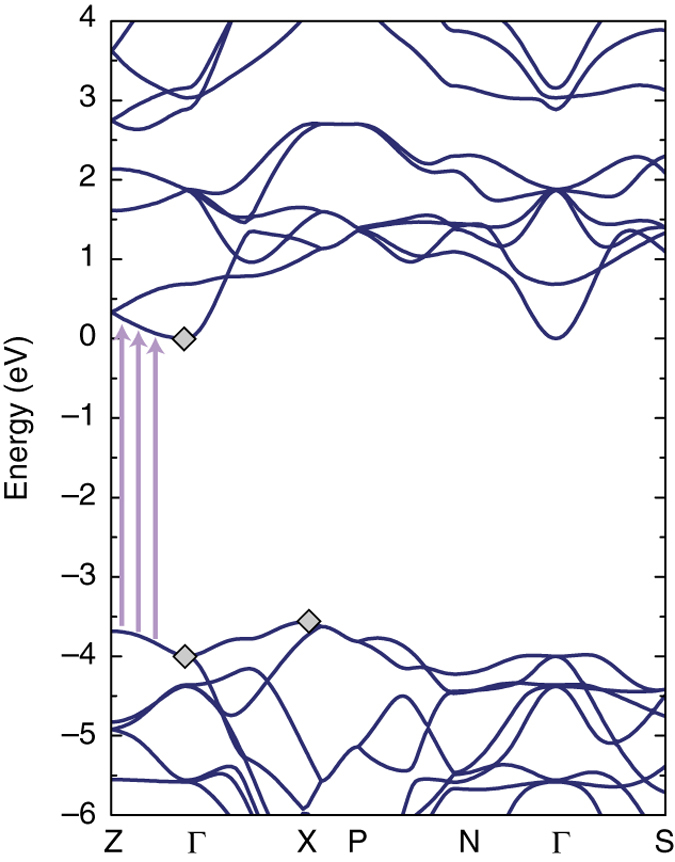
Calculated electronic structure. Electronic band structure of pristine anatase TiO_2_ in the first BZ. The *lines* indicate the DFT calculations corrected by the GW values. *Grey diamond dots* indicate the values obtained with the GW corrections at the Γ and X points, while the values along the high symmetry directions are obtained by correcting with linearly interpolated values

To rationalise our experimental results, we perform extensive ab initio calculations, including for the first time the effects of a finite doping and of the electron–phonon coupling on both the electronic and optical response of anatase TiO_2_. Combined with highly converged results for the pristine crystal, such calculations allow us to assess the role of doping and temperature via a more realistic model of the material, and to establish a direct comparison with the ARPES and SE data. First, we calculate the pristine anatase TiO_2_ GW band structure within the frozen lattice approximation. Figure 5 shows the complete band structure of pristine anatase TiO_2_. *Grey diamonds* denote the values obtained within GW for the VB and CB at the Γ and X points. These are also displayed in Fig. 3b for a direct comparison with the ARPES data. The overall agreement is good, but the theoretical gap values are higher by ~100 meV. This discrepancy can be caused by the effects of the doped electron density in the experimental data and/or the presence of strong electron–phonon interaction in anatase TiO_2_. In the following, we address the relevance of these separate effects.

In general, in the presence of doping, two competing effects contribute to changing the electronic gap of an insulator, resulting in an overall redshift or blueshift depending on which effect dominates. The CB filling gives rise to a blueshift, while BGR (with the system becoming slightly metallic) is responsible for a redshift as a result of exchange and correlation effects. To address the impact of the doped carrier density on the electronic band structure, the GW theoretical framework has been extended to the case of uniformly doped anatase TiO_2_. We observe that the change in the electronic direct gap at the Γ point is marginally affected by doping a uniform excess electron density for *n* ≤ 10^20^ cm^−3^. The calculated GW gaps (both direct and indirect gap) are similar to the pristine anatase TiO_2_ case, displaying only a slight increase well below the computational resolution (20 meV). This also implies that the dominant effect in anatase TiO_2_ for the considered doping range is the CB filling. These results are consistent with the experimental ARPES data of Supplementary Fig. 3 and show that the electronic gap of doped samples delivers a suitable value of the gap of pristine anatase TiO_2_. For this reason, hereafter, we refer only to the results of the GW calculations in the pristine sample. We will later show that the same holds for the optical response, as the position and shape of peak I in the calculated dielectric function do not change for *n* = 10^19^ cm^−3^. We therefore rule out the doped electron density as origin of the discrepancy between the experimental ARPES data and the calculations.

Thus, it is crucial to estimate the role of the electron–phonon interaction, which is known to be relatively strong in anatase TiO_2_^6, 27–32^. To this aim, we consider both zero point renormalization (ZPR) and electron–phonon coupling at finite temperature. For the former, we rely on recent theoretical data for rutile TiO_2_^33^, where a redshift of 150 meV was estimated for the electronic gap. Assuming a similar correction for anatase TiO_2_, we estimate a theoretical gap of 3.92 eV at zero temperature. To account for the electron–phonon coupling at finite temperature, frozen-phonon GW calculations for 20 and 300 K are performed. Specifically, we calculate the electronic bandgap when the ions in the primitive unit cell are displaced according to the eigenvectors of the longitudinal optical E_u_ and A_2u_ normal modes^34^, which are the ones most strongly coupled to the electronic degrees of freedom^6, 28^. We additionally consider the effect of lattice thermal expansion, which is also a source of renormalization for the quasiparticle gap. We find that the combined effect of the lattice expansion and electron–phonon coupling leads to a net blueshift of 30 to 50 meV at 300 K, while the shift is negligible at 20 K. Hence, at low temperature, the change in the electronic bandgap of anatase TiO_2_ is only due to the ZPR. This leads to a theoretical value of the direct bandgap of 3.92 eV, which is in excellent agreement with the experimental value of 3.97 ± 0.03 eV.

The complete GW band structure in Fig. 5 further yields important information about the Γ-Z direction, which is crucial for understanding the optical transitions of the material. The CB and VB dispersions between Γ and Z are nearly parallel, providing a large joint density of states for the optical transitions (shown as *violet arrows*). As discussed below, this peculiar dispersion leads to the intense excitonic transitions observed in the optical absorption spectrum.

**Fig. 6:**
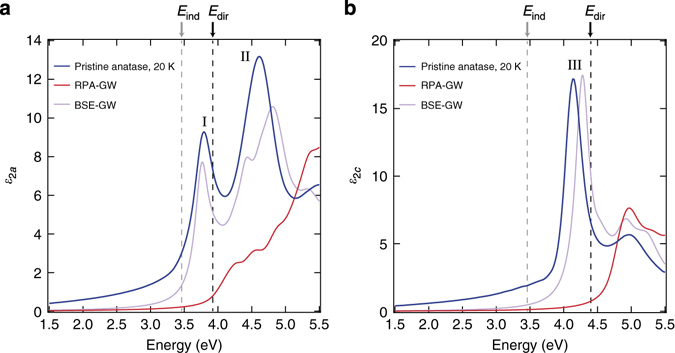
Calculated optical spectra of pristine anatase TiO_2_. **a**, **b** Comparison between the spectra measured at 20 K on the pristine anatase TiO_2_ single crystal and those obtained from frozen-lattice ab initio calculations for pristine anatase TiO_2_. The experimental data are reported in *blue*, the calculated spectra in the RPA-GW scheme in *red* and the calculated spectra in the BSE-GW scheme in *violet*. The quasiparticle indirect gap *E*_ind_ = 3.46 eV is indicated by a *dashed grey vertical line*; the direct gaps *E*_dir_ = 3.92 eV (at the Γ point) for **E** ⊥ c and *E*_dir_ = 4.40 eV (coincident with the onset of the RPA-GW) for **E** ǁ c are indicated by *dashed black vertical lines* in panel **a** and **b**, respectively

To identify the microscopic nature of the optical excitations, we calculate ε_2_(*ω*) for both pristine and doped anatase TiO_2_ at zero temperature. The effects of electron–phonon interactions are also included via frozen-phonon calculations at 20 and 300 K. The results for the pristine crystal at zero temperature, with and without many-body electron–hole correlations, are shown in Fig. 6a,b. The optical spectra in the uncorrelated-particle picture (*red lines*) are obtained within the random-phase approximation (RPA) on top of GW, while the many-body optical spectra (*violet lines*) are calculated by solving the Bethe–Salpeter Equation (BSE) as implemented in the BerkeleyGW code^35^ (*see* Methods and Supplementary Note 4). The inclusion of many-body effects^10–13^ yields an excellent agreement with the SE spectra. For **E** ⊥ c, the sharp absorption maximum at 3.76 eV is very close to feature I (3.79 eV) and well below the direct VB-to-CB optical transition evaluated at the independent particle level (3.92 eV, *red trace*). A second peak at 4.81 eV clearly corresponds to the experimental peak II (4.61 eV). For **E** ǁ c, the intense optical peak at 4.28 eV is easily assigned to the experimental peak III (4.13 eV). The BSE calculations performed for doped anatase TiO_2_ at concentrations of *n* = 10^19^ cm^−3^ and *n* = 10^20^ cm^−3^ (*see* Supplementary Note 6 and Supplementary Fig. 13) show a negligible effect on the considered optical peaks, supporting our SE data (Fig. 4a). The insensitivity of the energy of peak I with doping concentration suggests that effects of long-range Coulomb screening and BGR perfectly compensate each other for this transition, even at high doping levels.

We also investigate the role of lattice thermal expansion and electron–phonon interaction in the absorption spectra, by carrying out frozen-phonon BSE simulations, described in Supplementary Note 7. The position of the excitonic peak I at 20 K shows a negligible shift with respect to the zero temperature value and blueshifts by 75 meV due to the temperature increase from 20 to 300 K, in agreement with the experiment (Supplementary Fig. 6).

Finally, although exciton I is bound with respect to the direct electronic gap, it may retain a resonant character with respect to all phonon-mediated indirect transitions of the material. Hence, to account for possible effects originating from the indirect nature of the anatase TiO_2_ gap, we solve the BSE for a supercell, thus allowing for the coupling of electron and hole states via phonons with non-zero momenta. We find a negligible role of the phonon-mediated transitions in the exciton properties of anatase TiO_2_, beyond adding an Urbach tail at the lower energy side of peak I (*see* Supplementary Note 8 for details).

### Exciton isosurface and binding energy

**Fig. 7:**
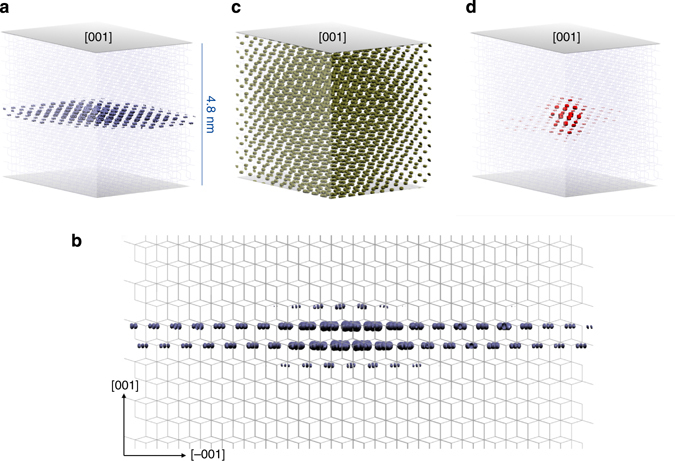
Wavefunctions of the fundamental charge excitations in anatase TiO_2_. Isosurface representation of the electronic configuration when the hole of the considered excitonic pair is localised close to one oxygen atom. The coloured region represents the excitonic squared modulus wavefunction. **a** Bound exciton I at 3.76 eV. **b** Side-view of the bound exciton I at 3.76 eV. **c** Resonance II at 4.37 eV. **d** Bound exciton III at 4.28 eV

The analysis in real and reciprocal space reveals very insightful information about the microscopic nature of the excitons. As far as the exciton associated with peak I is concerned, it extends two-dimensionally in a single (001) atomic plane. By fitting the exciton wavefunction with a 2D hydrogen model, we estimate the exciton Bohr radius around 3.2 nm, the 90% of the excitonic squared modulus wavefunction being contained within 1.5 nm (Fig. 7a,b). In addition, a reciprocal space analysis shows that this wavefunction is formed mainly by mixing of single-particle vertical transitions along the Γ-Z direction (Fig. 5). Due to the almost parallel CB and VB dispersion along Γ-Z, the electronic gap at Γ (coincident with the continuum absorption rise) is used as a reference to evaluate *E*_B_. Considering the renormalization of the electronic bandgap discussed above, we estimate a theoretical value of *E*_B_ = 160 meV at 20 K, in line with our measurements.

The energy, shape and reciprocal space contributions of peak II highlight its bulk-resonance character, most evident as its offset coincides with the independent particle-GW absorption rise. Figure 7c depicts the spatial distribution of the charge excitations associated with peak II, showing significant contributions from extended bulk states. Its electron wavefunction appears completely delocalised over many lattice constants around the hole. Hence, this peak is assigned to a resonant excitation that does not form a bound state. Carrying out a similar analysis for peak III, we conclude that it presents a mix of localised and bulk-resonant contributions, as the continuum onset in independent particle-GW undergoes an intensity enhancement. Different from peak I, the *k*-points contributing to this charge excitation are both located along the Γ-Z line and distributed in the whole BZ. Indeed, as shown in Fig. 7d, the linear combination of excitonic wavefunctions (with eigenvalues in the range 4.18–4.38 eV for **E** ǁ c) contributing to peak III leads to a fairly localised exciton in all three directions. By fitting only the bound contribution to the wavefunction with a 3D hydrogen model, we obtain an average radius of 0.7 nm, corresponding to less than two lattice constants in the plane. By including also the contributions from resonant states, the 90% of the exciton wavefunction square modulus is contained within 2 nm. Due to the mixed nature of exciton III, it is less straightforward to define and estimate its *E*_B_. Assuming the independent particle-GW onset at 4.40 eV for **E** ǁ c as the reference energy (marked as *E*_dir_ in Fig. 6b), we obtain *E*_B_~150 meV (in the frozen lattice scheme), which is still well above the standard *E*_B_ observed in bulk semiconductors. From this analysis, we conclude that excitons I and III, due to their *E*_B_ and spatial nature, retain an intermediate character between Frenkel and Wannier–Mott regimes. On the other hand, peak II corresponds to a resonance that does not form a bound exciton.

### Exciton physics in anatase TiO_2_ NPs

**Fig. 8:**
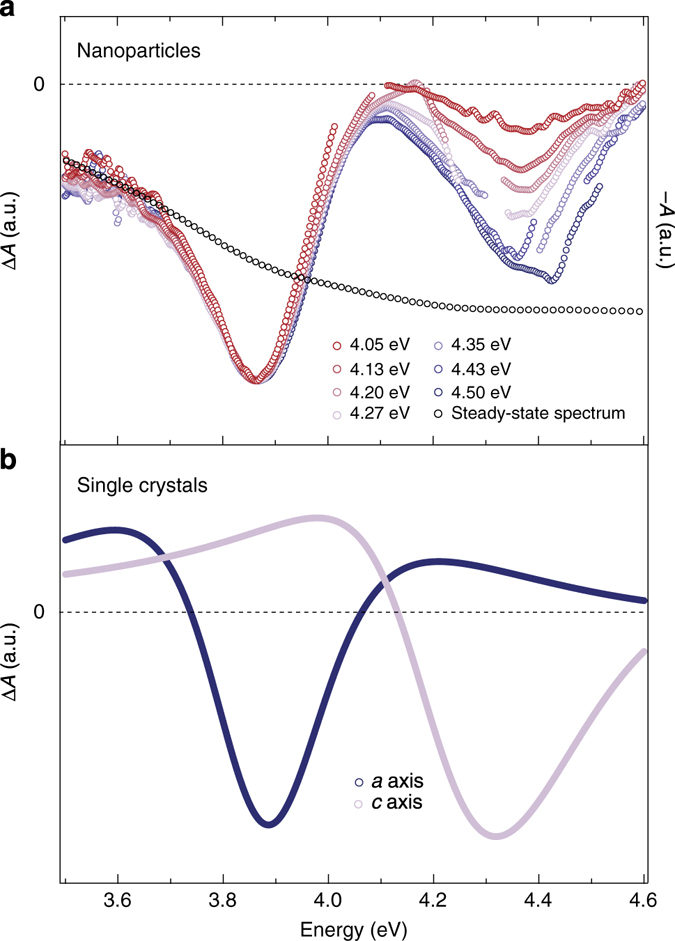
Ultrafast 2D UV spectroscopy of anatase TiO_2_ NPs and single crystals. **a** Normalised transient absorption (Δ*A*) spectra of RT colloidal solution of anatase TiO_2_ NPs at a fixed time-delay of 1 ps and for different pump photon energies (indicated in the figure). Each trace is normalised with respect to the minimum of the main feature at 3.88 eV. For comparison, the *black trace* shows the inverted steady-state absorption spectrum. **b** Δ*A* spectrum of RT anatase TiO_2_ single crystals along the *a*-axis and c-axis at a fixed time delay of 1 ps. For this experiment, the pump photon energy is 4.40 eV

The above description dealt with bulk single crystals of anatase TiO_2_, but in most applications^1–3^, defect-rich samples are used at RT and ambient pressure (*e.g.*, NPs or mesoporous films), and one may therefore question the validity of the above conclusions to the actual systems used in applications. Indeed, one could expect that the carriers released at defects and the local electric fields generated by charged impurities would screen the Coulomb interaction in the exciton, leading to the cancellation of the binding forces. Moreover, strong exciton-defect and exciton-impurity scattering can also cause an extreme broadening of the exciton linewidth, hiding the characteristic exciton feature into the continuum of indirect interband excitations. These ideas are reinforced by the equilibrium absorption spectrum of colloidal anatase TiO_2_ NPs (of unknown doping), which does not show obvious signatures of excitonic transitions^36, 37^ (Supplementary Fig. 1 and *black trace* in Fig. 8a). However, this spectrum is also strongly affected by the scattering of the incident light and this can in turn affect the detection of the excitonic peaks. To circumvent this problem, we interrogate the system out-of-equilibrium via ultrafast 2D UV transient absorption spectroscopy^38^, since this technique subtracts the scattered light and provides a better contrast for resolving hidden features. It is applied here for the first time to solid samples, and it offers the capability to excite across the gap of a wide-gap material with tunable photon energy and probe in the same region with a broadband continuum. Typically, the exciton lineshapes can be identified through the pump-induced transparency of the excitonic peak, referred to as exciton bleaching. This nonlinear optical process is intrinsically related to a many-body phenomenon: Its manifestation depends not only on the final-state interactions of electron and hole involved in the excitonic state but also on the interaction with all other particles in the material, which can contribute to screening or blocking the excitonic transition^39, 40^.

We carry out 2D UV spectroscopy of doped single crystals (*n*~0 cm^−3^, *n* = 2 × 10^17^ cm^−3^ and *n* = 2 × 10^19^ cm^−3^) in transient reflectivity (Δ*R*/*R*) and of a colloidal solution of NPs in transient absorption (Δ*A*). For the single crystals, we retrieve the anisotropic Δ*R*/*R* dynamics along the *a*-axis (Supplementary Figs. 8a,b) and *c*-axis (Supplementary Figs. 8c,d and 9) in a broad UV range, using a pump photon energy of 4.40 eV. Subsequently, we extract Δ*A* from the Δ*R*/*R* response (*see* Supplementary Note 3). Figure 8a,b, respectively show the normalised Δ*A* of anatase TiO_2_ NPs in aqueous solution and of doped anatase TiO_2_ single crystals (*n* = 2 × 10^19^ cm^−3^), as a function of the probe photon energy, at a time delay of 1 ps and for different pump photon energies. Figure 8a exhibits a negative (*bleach*) signal over the entire probe range, displaying two prominent features at 3.88 and 4.35 eV. While the former excitation is present at all pump photon energies, the latter becomes more prominent for pump photon energies beyond 4.10 eV, i.e., in correspondence with the threshold for accessing the *c*-axis optical response. Both features have a full-width at half maximum of 300 ± 20 meV. Figure 8b shows the derived Δ*A* along the *a*-axis and *c*-axis for the doped single crystal, under 4.40 eV excitation and at a time delay of 1 ps. In these separate polarisation channels, two negative features appear at 3.88 and 4.32 eV, similarly to the Δ*A* spectra of NPs. These energies match those of the excitonic peaks I and III in the equilibrium absorption spectrum along the *a*-axis and *c*-axis (Supplementary Fig. 4c,d, note that the values of the steady-state absorption peaks slightly differ from the peaks in the dielectric function). Hence, this analysis leads us to disentangle the *a*-axis and *c*-axis contributions in the Δ*A* response of the NPs. Both excitons are found to appear in the latter due to the random orientation of NPs in solution. Therefore, we conclude that the excitonic features are also present in the equilibrium absorption spectrum of anatase TiO_2_ NPs (Supplementary Fig. 1), but they are obscured by the strong light scattering. This highlights the ability of ultrafast 2D deep-UV spectroscopy to reveal hidden features in the spectral response of wide-gap materials.

## Discussion

Our extensive study allows us to demonstrate the stability of bound excitons in anatase TiO_2_. Excitonic effects have been thought to be weak in this material, due to its large static dielectric constant (*ε*_S_~22/45)^34, 41^. Moreover, due to a smaller effective mass^42^, one would expect the electron–hole interaction to be even weaker than that in rutile TiO_2_, in which *E*_B_ has been estimated ~4 meV^43, 44^. However, the presence of a large *ε*_S_ is not a sufficient condition to prevent the formation of excitons in materials, as the screening of the electron–hole interaction should rigorously take into account the momentum- and energy-dependence of the dielectric constant^27^. Therefore, it is the combination of the electronic structure and the nature of the screening that determines the existence of bound excitons in materials. The degree of excitonic spatial delocalisation is instead influenced by the crystal structure, as the packing of the polyhedra containing the atoms involved in the excitonic transitions is related to the details of the band structure. These observations rationalise well the exciton physics in titanates.

A necessary condition for a many-state transition to occur and for a bound collective excitonic state to form is that the electron and hole group velocities are the same, i.e., that the gradients of the lowest CB and the highest VB are identical in a specific portion of the BZ^27^. As a matter of fact, the band edge states of anatase TiO_2_ (Fig. 5) and SrTiO_3_^45, 46^ are parallel in extended portions of the BZ, which contribute density of states to the collective transition associated with bound excitonic species (*E*_B_~180/220 meV)^11, 47^. In contrast, the band structure of rutile TiO_2_ is not characterised by such extended portions with similar electron and hole group velocities^11^, thus resulting in *E*_B_~4 meV^43, 44^.

Concerning the spatial distribution of the excitons in SrTiO_3_ and TiO_2_, since all the bound excitonic transitions are predicted to involve O 2*p* and Ti 3*d* (*t*_2*g*_) states^11, 12, 47^, one should consider the role of different TiO_6_ octahedra packing. In SrTiO_3_, the unit cell is built on a distorted perovskite structure, thus providing a high degree of coordination among neighbouring TiO_6_ octahedra. As a consequence, the single-particle band states involved in the excitonic transition undergo pronounced dispersion and the strongly bound exciton predicted in SrTiO_3_ retains a highly delocalised nature similarly to a Wannier–Mott exciton^47^. In TiO_2_, both the anatase and rutile polymorphs are also built on a network of coordinated TiO_6_ octahedra, but they significantly differ in their structural properties. In rutile, each distorted TiO_6_ octahedron is connected to ten neighbouring ones, sharing a corner or an edge. In anatase, the coordination of the TiO_6_ octahedra is less compact and each octahedron is coordinated only with eight neighbouring ones. This seemingly tiny difference has in turn profound effects on the spatial properties of the elementary charge excitations: While in rutile the weakly-bound excitons are Wannier–Mott-like, in anatase the bound exciton is confined to the (001) plane^11^. More specifically, it is the chain-like structure of anatase TiO_2_ that leads to unique characteristics of the band structure and hinders the delocalisation in 3D of the bound exciton over many unit cells. Indeed, the band dispersion along the Γ-Z direction is rather flat, thus implying a high degree of localisation for the excitonic state along the *c*-axis. This almost 2D wavefunction also contributes to enhance the exciton *E*_B_, in a way similar to the low-dimensional effect in semiconductor quantum structures^40^. Under these conditions, according to the 2D hydrogen model, *E*_B_ is four times larger than for a 3D exciton. With *E*_B_ exceeding the highest energy of the longitudinal optical phonons (i.e., 108 meV in anatase TiO_2_)^34^, the ionic (polaronic) contribution to the screening of the Coulomb interaction is strongly suppressed^27^. As a consequence, the total screening reduces to the pure contribution of the background valence electrons, embodied by the rather small dielectric constant at optical frequencies *ε*_opt_ ~ 6/8. This weak screening in turn reduces the exciton Bohr radius on the (001) plane (~3.2 nm).

Following these arguments, the 2D wavefunction of exciton I in anatase TiO_2_ represents a peculiar and fascinating object. Indeed, in recent years, several 2D excitons were reported in materials such as, hexagonal boron nitride^48^ and transition metal dichalcogenides^49–51^, which are layered 2D systems held together by van der Waals forces to form a 3D lattice. The situation of anatase TiO_2_ is radically different in that a genuine 3D material exhibits a 2D exciton wavefunction on a specific lattice plane.

From the point of view of devices, the newly discovered excitons may provide a significant source of optical nonlinearity, paving the way to the development of electro-optical or all-optical switches in the UV. Also engineered nanostructures exposing a large percentage of (001) facets can be useful in guiding the energy at the nanoscale in a selective way^52–54^. Finally, due to the important contribution that phonons have on the exciton width and lineshape, we expect that the optical properties of anatase TiO_2_ can be effectively altered by tuning the exciton–phonon coupling, e.g., through the applications of mechanical strain. In this regard, new insights from many-body theory will be crucial for evaluating the transport of these excitonic species, their coupling to the vibrational degrees of freedom and their reaction to various external stimuli.

## Methods

### Single crystal growth and characterisation

High-quality single crystals of anatase TiO_2_ were produced by a chemical transport method from anatase powder and NH_4_Cl as transport agent, similar to the procedure described in ref. 55. In detail, 0.5 g of high-purity anatase powder were sealed in a 3 mm thick, 2 cm large and 20 cm long quartz ampoule together with 150 mg of NH_4_Cl, previously dried at 60 °C under dynamic vacuum for one night, and 400 mbar of electronic grade HCl. The ampoules were placed in a horizontal tubular two-zone furnace and heated very slowly to 740 °C at the source, and 610 °C at the deposition zone. After 2 weeks, millimetre-sized crystals with a bi-pyramidal shape were collected and cut into rectangular bars (typically 0.8 × 0.6 × 0.15 mm^3^). Copper-doped anatase TiO_2_ single crystals were obtained by annealing raw anatase single crystals in O_2_ at 700 °C for 6 days in the presence of Cu vapours. The pristine form of anatase TiO_2_ was instead obtained by annealing the raw anatase TiO_2_ crystals at 700 °C for 10 days under 950 mbar of CO. The doping levels of the raw, copper-doped and pristine crystals were determined via ARPES or transport measurements to be *n* = 2 × 10^19^ cm^−3^, *n* = 2 × 10^17^ cm^−3^ and *n *~ 0 cm^−3^, respectively.

### NPs synthesis and characterisation

Anatase TiO_2_ NPs were prepared by the sol–gel method^56^. The synthesis was carried out in a glove box under argon atmosphere. Titanium isopropoxide (Sigma Aldrich, 99.999% purity) was used as precursor and mixed with 10 ml of 2-propanol. This mixture was added dropwise under vigorous stirring to cold acidic water (2 °C, 250 ml H_2_O, 18 MΩ, mixed with 80 ml glacial acetic acid, final pH 2). At the beginning, the mixture looked turbid, but after stirring it in an ice bath for 12 h, it became transparent as the amorphous NPs were formed. The mixture was then peptized at 80 °C for about 2 h until the liquid turned into a transparent gel. The gel was autoclaved at 230 °C for 12 h. During this process, the previous amorphous sample became denser and underwent a phase transition, resulting in anatase TiO_2_ NPs. After the autoclave, the NPs precipitated to the bottom of the container. They were separated from the supernatant and added to 100 ml acidic water (pH 2) to obtain a white colloidal solution with a final concentration of ca. 337 mM. In refs 57, 58, we reported the details of the sample characterisation by means of X-ray diffraction and transmission electron microscopy. These techniques enabled us to demonstrate the good quality of the anatase phase and the spherical shape (with an average diameter of approximately 25 nm) of the NPs. The doping of the NPs was not estimated and, thus, it is unknown. The steady-state absorption spectrum of the colloidal solution of anatase TiO_2_ was recorded at RT using a commercial UV-VIS-NIR spectrometer (Shimadzu, UV-3600). Before measuring the absorption spectrum of the sample, a reference spectrum of the pure solvent (acidic water, pH 2) was taken to check its transparency in the investigated spectral range.

### Angle-resolved photoemission spectroscopy (ARPES)

The ARPES measurements were performed at the Electronic Structure Factory endstation on beamline 7.0.1 at the Advanced Light Source, Berkeley, CA, USA. A raw anatase TiO_2_ single crystal was polished and cleaned in a buffered 5% fluoric acid solution before introducing it into the ultra-high vacuum system (<10^−10^ mbar). The crystal was annealed in 35 mbar of oxygen at 400 °C for 30 min before the ARPES experiments. The ARPES measurements were performed at a photon energy *hv* = 128 eV and with an energy resolution of 30 meV.

### Spectroscopic ellipsometry (SE)

Using SE, we measured the complex dielectric function of the sample, covering the spectral range from 1.50 to 5.50 eV. The measurements were performed using a Woollam VASE ellipsometer. The single crystals with *n *~ 0 cm^−3^ and *n* = 2 × 10^19^ cm^−3^ were polished along a (010)-oriented surface and mounted in a helium flow cryostat, allowing measurements from RT down to 10 K. When at cryogenic temperatures, the measurements were performed at <10^−8^ mbar to prevent measurable ice-condensation onto the sample. Anisotropy corrections were performed using standard numerical procedures^59^ and diffraction effects at low frequency were accounted for using the procedure developed in ref. 60. The SE data have been further corrected to account for the surface roughness of the single crystal, which was estimated around 0.9 nm by means of atomic force microscopy (AFM). Supplementary Fig. 2a,b shows two images taken under the AFM for the *n *~ 0 cm^−3^ crystal. The average surface roughness of the polished surfaces was ~0.9 nm. The steady-state reflectance at 100 K was derived from the measured dielectric function to be compared to previous normal-incidence reflectivity measurements (Supplementary Note 1 and Supplementary Fig. 4a,b). The precision of the Kramers–Kronig analysis used to treat the normal-incidence reflectivity data was also tested (Supplementary Fig. 5).

### Ultrafast 2D UV spectroscopy

The ultrafast optical experiments were performed using a novel setup of tunable UV pump and broadband UV probe, described in detail in ref. 38. A 20 kHz Ti:Sapphire regenerative amplifier (KMLabs, Halcyon + Wyvern500), providing pulses at 1.55 eV, with typically 0.6 mJ energy and around 50 fs duration, pumped a noncollinear optical parametric amplifier (NOPA) (TOPAS white-Light Conversion) to generate sub-90 fs visible pulses (1.77–2.30 eV range). The typical output energy per pulse was 13 μJ. Around 60% of the output of the NOPA was used to generate the narrowband pump pulses. The visible beam, after passing through a chopper, operating at 10 kHz and phase-locked to the laser system, was focused onto a 2 mm thick barium borate (BBO) crystal for nonlinear frequency doubling. The pump photon energy was controlled by the rotation of the crystal around the ordinary axis and could be tuned in a spectral range up to ~0.9 eV (~60 nm) wide. The typical pump bandwidth was 0.02 eV (1.5 nm) and the maximum excitation energy was about 120 nJ. The pump power was recorded on a shot-to-shot basis by a calibrated photodiode for each pump photon energy, allowing for the normalisation of the data for the pump power. The remaining NOPA output was used to generate the broadband UV probe pulses with ~1.3 eV (~100 nm) bandwidth through an achromatic doubling scheme. Pump and probe pulses, which have the same polarisation, were focused onto the sample, where they were spatially and temporally overlapped. The typical spot size of the pump and the probe were 100 and 40 μm full-width at half-maximum, respectively, resulting in a homogeneous illumination of the probed region.

This setup could be used either in a transmission or in a reflection configuration. The anatase TiO_2_ single crystals were studied by detecting their transient reflectivity (Δ*R*/*R*) upon photoexcitation, while the NPs were investigated by recording their transient absorption (Δ*A*). In the case of the measurements on the anatase TiO_2_ single crystals, the specimens were mounted on a rotating sample holder, in order to explore the Δ*R*/*R* response along the desired crystalline axis. The measurements were performed on the three different classes of samples (*n *~ 0 cm^−3^, *n* = 2 × 10^17^ cm^−3^ and *n* = 2 × 10^19^ cm^−3^, *see* Supplementary Note 2 and Supplementary Fig. 7). The portion of the probe beam reflected by the surface of the crystal was detected, and the time evolution of the difference in the UV probe reflection with and without the pump pulse reconstructed. All the experiments were performed at RT. Concerning the measurements on the anatase NPs, the sample consisted of anatase TiO_2_ NPs dispersed in an aqueous solution (20% acetic acid and 80% water) to avoid interparticle charge-transfer. The colloidal solution circulated into a 0.2 mm thick quartz flow-cell to prevent photo-damage and its concentration was adjusted to provide an optical density of approximately 0.4. The probe was measured after its transmission through the sample and its detection synchronized with the laser repetition rate. The difference of the probe absorption with and without the pump pulse was measured at different time delays between the pump and the probe, by means of a motorised delay line in the probe path. After the sample, the transmitted/reflected broadband probe beam was focused in a multi-mode optical fibre (100 μm), coupled to the entrance slit of a 0.25 m imaging spectrograph (Chromex 250 is). The beam was dispersed by a 150 g mm^−1^ holographic grating and imaged onto a multichannel detector consisting of a 512 pixel CMOS linear sensor (Hamamatsu S11105, 12.5 × 250 μm pixel size) with up to 50 MHz pixel readout, so the maximum read-out rate per spectrum (almost 100 kHz) allowed us to perform shot-to-shot detection easily. The described experimental setup offered a time resolution of 150 fs.

### Ab initio calculations—pristine anatase TiO_2_

Many-body perturbation theory at the level of the GW and the BSE^61–63^ was employed to compute the band structure and the dielectric response of bulk anatase TiO_2_. The GW and BSE calculations were performed on-top of eigenvalues and eigenfunctions obtained from DFT. We used the planewave pseudopotential implementation of DFT as provided by the package Quantum Espresso. GW and BSE calculations were performed with the BerkeleyGW package^35^. We also used the GW + BSE Yambo^64^ implementation to verify that the results of our calculations are code independent.

The DFT calculations were performed using the generalized gradient approximation (GGA) as in the Perdew–Burke–Ernzerhof (PBE) scheme for the exchange-correlation functional. The Ti norm-conserving pseudopotential was generated in the Rappe–Rabe–Kaxiras–Joannopoulos scheme^65^, including semicore 3*s* and 3*p* states. While standard structural and electronic quantities are already converged in DFT with an energy cutoff of 90 Ry, the energy cutoff used here was raised to 160 Ry to properly include the high number of bands necessary to reach convergence for the many-body evaluated properties. Bulk anatase TiO_2_ was modelled on a body-centred tetragonal lattice containing two Ti atoms and four O atoms (primitive cell) with lattice parameters (optimised at the PBE level) *a* = *b* = 3.79 Å and *c* = 9.66 Å. The experimental lattice constants at RT are *a* = *b* = 3.78 Å and *c* = 9.51 Å. Scaling these parameters to zero temperature via a linear extrapolation^66^ of the temperature dependence of the lattice constant at high temperature, appearing in ref. 67, yields *a* = *b* = 3.78 Å and *c* = 9.49 Å.

The ground state electronic density is properly described with a coarse 4 × 4 × 4 *k*-point grid for sampling of the BZ. The GW quasiparticle corrections to the DFT eigenvalues were performed at the one-shot level of theory (G_0_W_0_). For the computation of the polarizability and inverse dielectric matrices in BerkeleyGW, we employed a total of 2474 CBs and G-vectors with kinetic energies up to 46 Ry, whereas the self-energy operator was computed using 2472 unoccupied bands and a G-vector cut-off energy of 46 Ry and 160 Ry for the screened and bare Coulomb matrices, respectively. The coarse 4 × 4 × 4 *k*-point grid sampling is sufficient for the description of the quasiparticle corrections, while a high number of bands is mandatory to get a proper description of screening effects and many-body corrections. The electronic band structure was finally obtained by interpolating GW corrections on top of a more refined DFT calculation with a 16 × 16 × 16 grid. The fully converged BSE results shown in the main text were obtained with BerkeleyGW. We used a shifted grid with up to 16 × 16 × 16 *k*-points (4096 irreducible *k*-points). The six lowest CBs and six topmost VBs were included to solve the excitonic Hamiltonian. The results (shown in Supplementary Figs. 10a,b, 11 and 12) were code-independent, as verified by comparing the BerkeleyGW results with those obtained with the Yambo code at the same level of convergence^11^. All results shown in this paper were obtained with the resonant part of the excitonic Hamiltonian (inclusion of the antiresonant part does not lead to significant changes). Spin-polarised calculations were performed to highlight possible dark excitons due to triplet excitations but no measurable differences with respect to the spin-restricted results were obtained. The novelty of these calculations compared to the results reported in literature is described in Supplementary Note 5.

### Ab initio calculations—doped anatase TiO_2_

Within the same theoretical framework used for pristine anatase TiO_2_, we performed calculations for the case of uniformly doped anatase TiO_2_, to verify computationally that the influence of doping on both the electronic gap and optical response can be disregarded. In Supplementary Note 6, we report the results for two cases of uniform excess electron density *n* = 10^19^ cm^−3^ and *n* = 10^20^ cm^−3^.

### Ab initio calculations—Electron–phonon coupling

To estimate the role of the electron–phonon coupling in the electronic and optical properties of anatase TiO_2_, we performed frozen phonon DFT + GW + BSE calculations by separately displacing the ions in the primitive unit cell according to the eigenvector of the longitudinal optical E_u_ and A_2u_ normal modes^34^, which are those possessing the stronger coupling with the electronic degrees of freedom^6, 28^. The displacement of atom *j* was calculated from the harmonic oscillator mean square displacement at 300 K according to

1 $$\left \langle {\left| {{u_{\rm{j}}}\left( t \right)} \right|^2} \right\rangle \frac{{\hbar \left( {1 + 2{n_{{\rm{BE}}}}} \right)}}{{2{m_{\rm j}}\omega }}$$

where ${n_{{\rm{BE}}}} = {\left( {{{\rm e}^{\hbar \omega /{k_{\rm{B}}}T}} - 1} \right)^{ - 1}}$ is the Bose-Einstein statistical occupation factor, T is the temperature, *k*_B_ is the Boltzmann constant, *m*_j_ is the atomic mass and *ω* is the phonon frequency. The results of these calculations are reported in Supplementary Note 7.

### Ab initio calculations—Indirect excitations

Anatase TiO_2_ is an indirect bandgap material with a minimum indirect gap amounting to 3.61 eV, according to our calculations without ZPR corrections. This gap is smaller than the optical gap we obtained at the BSE level of theory (3.76 eV). As discussed in the main text, exciton I is bound with respect to the direct gap. Despite this, the exciton could also be considered as resonant with respect to all phonon-mediated indirect transitions. In the frozen-phonon calculations for a single TiO_2_ unit cell, the BSE includes only coupling of direct electron–hole transitions with phonons at the Γ point, and hence, possible effects originating from the indirect nature of the material would not be accounted for a way to incorporate those effects is to perform BSE calculations for a large TiO_2_ supercell, where both the indirect gap and direct gap are folded into the $\tilde \Gamma$ point of the supercell. In such a calculation, frozen atom displacements can couple electron and hole states with different *k* values in the original sampling of the first BZ via phonons with nonzero *q*-vectors. The results of these calculations are reported in Supplementary Note 8. We have recently become aware of ref. 68, in which a one-shot method to compute the indirect contributions to the absorption tail using supercell methods has been proposed.

### Data availability

The data that support the findings of this study are available from the corresponding authors upon reasonable request.

## Electronic supplementary material

Supplementary Information Supplementary Figures, Supplementary Notes and Supplementary References

Peer Review File Reviewer reports and authors' response from the peer review of this Article at Nature Communications
