# Supplementary material for: Strongly bound excitons in anatase TiO2 single crystals and nanoparticles
Source: Nat Commun. 2017 Apr 13;8:13. doi: 10.1038/s41467-017-00016-6 (PMC5432032; doi:10.1038/s41467-017-00016-6)
Supplement: Supplementary file 1 — Supplementary Figures, Supplementary Notes and Supplementary References [file 41467_2017_16_MOESM1_ESM.pdf]

Additional experimental results

Supplementary Note 1: Steady-state spectroscopic ellipsometry

Comparison with previous experiments. Our spectroscopic ellipsometry (SE) measurements are performed on two classes of (010)-oriented single crystals, namely pristine anatase ($n \sim 0$ cm^{-3}) and n-doped anatase ($n = 2 \times 10^{19}$ cm^{-3}). The reflectivity response derived from SE on the pristine (violet lines) and n-doped (blue lines) single crystals is plotted in Supplementary Figs. 4a-b together with the data of Ref. 1 (red lines). The reflectivities for light polarized along the a- and c-axis of the crystals are shown in Supplementary Figs. 4a and 4b, respectively. To establish a direct comparison with the normal-incidence reflectivity data of Ref. 1, all reported traces have been measured at 100 K. We observe the overall agreement of our results with the ones reported in literature, with slight shifts in the reflectivity peak energies. However, given the sharpness of the reflectivity lineshapes, we can also deduce that the quality of our pristine crystal is much higher than the one employed in Ref. 1. Indeed, the reflectivity spectrum of the latter is closer to the response measured on our n-doped crystal, which has a large percentage of oxygen vacancies. Furthermore, we note that the two different energy scales used in Ref. 1 to display the data of the dielectric function for $\mathbf{E} \perp$ c and $\mathbf{E} \parallel$ c has led to an error of digitalization by early computational works, which has propagated in the literature[2–5] on anatase TiO_2.
Supplementary Figs. 4c-d display the normalized absorption spectra of our anatase TiO_2 single crystals at 300 K. The a- and c-axis responses are respectively shown in Supplementary Figs. 4c and 4d. Here, our interest is to reveal the robustness of the excitonic peaks even at room temperature (RT) and to highlight the role played by the presence of oxygen vacancies in the doped sample. Hence, for this purpose, the data are normalized with respect to the lowest direct exciton peaks. We observe that in both the a- and c-axis absorption spectra the exciton peak energies are not renormalized by the presence of oxygen vacancies, while the linewidth becomes broader in the n-doped crystal.
Figures 5a and 5b display $\epsilon_1(\omega)$ and $\epsilon_2(\omega)$ calculated by applying the Kramers-Kronig (KK) analysis on the reflectivity spectrum at RT. This technique represents a valuable test to evaluate the consistency with our original $\epsilon_1(\omega)$ and $\epsilon_2(\omega)$ values. We observe that the effect of the KK

transformation on both the a- and c-axis $\epsilon_2(\omega)$ is to modify the lineshape and the intensity of the peaks and to change the spectral weight in the Urbach tail. This behaviour shows that the KK analysis, even performed on the broadest possible spectral range, is not a precise approach to treat the normal-incidence reflectivity data.

Many-body effects. In the case of the n-doped ($n = 2 \times 10^{19}$ cm^{-3}) anatase TiO_2 crystals, the presence of an excess electron density at the Γ point of the CB may give rise to a variety of effects having a profound impact on both the single-particle and the two-particle excitation spectra. According to their origin, these effects can be distinguished between those involving single-particle states (phase-space filling) and those that can be attributed to many-body interactions among the doped carriers (long-range Coulomb screening and band gap renormalization, BGR). Phase-space filling arises because of the Pauli exclusion principle, which applies to the electrons and holes constituting the excitons. This produces a finite exclusion volume in phase-space for each exciton. As a consequence, the VB-to-CB transition probability is reduced, which is evidenced by a reduction of oscillator strength of the excitonic transition in the optical spectra. On the other hand, many-body interactions alter the exciton energy and composition by affecting the underlying electronic states. This occurs via the direct and exchange Coulomb interactions of electrons and holes, which at high plasma density provide additional screening channels. First, long-range free-carrier screening modifies the exciton Coulomb potential $\phi \sim \frac{e}{4\pi\epsilon r}$ through a multiplicative factor $e^{-r/\lambda}$, where λ is the Debye length. Substantial screening occurs when the Debye length and the exciton radius become comparable. Second, the excess electrons also lead to BGR, *i.e.* a density-dependent shrinkage of the quasiparticle gap due to electron and hole self-energy corrections. As a result, the enhancement of the electronic screening leads to the simultaneous renormalization of both the exciton E_{B} and the electronic gap. In this scenario, the exciton absorption energy in the SE data is determined by the combination of the weakened Coulomb interaction and the BGR, the former inducing a blueshift and the latter a redshift of the exciton peak. The quantitative details of this compensation depend on both material and dimensionality but this arises as a general effect in many standard bulk semiconductors[6] and nanostructures.[7,8] In the low-density limit, they have been shown to cancel

to first order.[9] Relying on this discussion, we can provide a deeper interpretation of the changes in the low-temperature dielectric function of anatase TiO_2 upon electron doping (Fig. 4). The redistribution of spectral-weight can be attributed to the combination of phase-space filling and defect-induced in-gap absorption. The enhanced broadening of the exciton lineshapes can be directly associated with a modification of the exciton lifetime, due to the combination of long-range Coulomb screening and exciton-defect scattering. Finally, the insensitivity of the exciton energies to the effective doping level suggests that the long-range Coulomb screening and the BGR perfectly compensate each other even at high doping levels. This idea is reinforced by our *ab initio* calculations (see Supplementary Note 6), which reveal that doping-induced many-body effects in anatase TiO_2 play a marginal role even at high carrier densities.

Supplementary Note 2: Ultrafast transient reflectivity of single crystals

Femtosecond transient reflectivity experiments are performed on three different classes of (001)- and (010)-oriented single crystals, with $n \sim 0$ cm^{-3}, $n = 2 \times 10^{17}$ cm^{-3} and $n = 2 \times 10^{19}$ cm^{-3}.

In a first experiment, the $\Delta R/R$ signal is monitored in a broadband UV range (3.75 - 4.35 eV) for the three classes of samples along the ab planes. Both pump and probe polarizations are set in a parallel configuration. An isotropic optical response is found when the (001)-oriented crystals are rotated about the c-axis. The pump energy is 4.10 eV, in order to selectively perturb the spectral region above the first excitonic peak. Supplementary Figure 7a compares the transient spectrum obtained from the three specimens at a fixed time-delay of 6 ps. The $\Delta R/R$ spectrum shows an inversion point around 3.96 eV. The inflection that is present around 4.10 eV in the signal obtained from the sample with $n = 2 \times 10^{17}$ cm^{-3} is an artefact produced by the scattering of the pump beam in our spectrometer. A similar response can be found for all the temporal delays between pump and probe up to 1 ns. The main difference is displayed by the intensity of the signal at long time delays, since it depends on the rate of the carrier recombination process. Supplementary Figure 7b shows three temporal traces up to 100 ps probed around 3.82 eV, in which this effect is clearly visible. The decay of the nonequilibrium

signal in the strongly n-doped crystal is faster than the other responses, since the increased density of in-gap states facilitates charge carrier recombination across the bandgap. The detailed analysis of the kinetics will be subject of a separate publication.

A second set of experiments is performed on (001)- and (010)-oriented n-doped ($n = 2 \times 10^{19}$ cm^{-3}) single crystals of anatase TiO_2, in order to access the anisotropic dynamics along the a- and c-axis. Supplementary Figures 8a and 8c display the $\Delta R/R$ maps of the a- and c-axis response for the (010)-oriented single crystal as a function of the probe photon energy and of the time delay between pump and probe. Although the kinetics are measured up to 1 ns, the two maps are displayed up to 10 ps. The a-axis response is measured upon photoexcitation at 4.40 eV, with the broadband probe covering the range 3.70 - 4.65 eV. These results can be also reproduced by polarizing the beams along the a-axis of the (001)-oriented samples. The higher-energy region of the spectrum does not evolve in time, remaining unaffected by the photoexcitation process. The c-axis response (Supplementary Fig. 8c) is obtained through the measurement of the (010)-oriented single crystal with a pump and probe polarizations set along the c-axis. Also in this case the pump energy is at 4.40 eV, but the probed range is shifted to 4.05 - 4.80 eV. The transient spectrum of Supplementary Fig. 8d strongly differs from the in-plane one, consisting of a negative contribution set around 4.28 eV and a tail extending to 4.60 eV in the high-energy range.

In a final experiment, both the pump (at 4.40 eV) and the probe (between 3.70 and 4.60 eV) beams are polarized along the c-axis of the $n = 2 \times 10^{19}$ cm^{-3} single crystal. The resulting $\Delta R/R$ map is displayed in Supplementary Fig. 9. This demonstrates the absence of c-axis spectral features at low energies and confirms the finding of the pump-probe experiment along the c-axis shown in Supplementary Figs. 8c-d. The difference in terms of intensity with respect to those measurements has to be attributed to the reduced pump intensity (of $\sim 1/3$) due to constraints in the generation of the broadband probe beam.

Data analysis and theory

Supplementary Note 3: Extracting ΔA from $\Delta R/R$ in single crystals

In our ultrafast measurements, we probe $\Delta R/R$ of anatase TiO_2 single crystals and ΔA of a colloidal solution of NPs. Thus, it is useful to analyze the time-resolved dynamics of the single crystals in terms of their transient absorption, in order to establish a link with the data on NPs. However, we recall that $\Delta R/R$ has a non-trivial relationship with both $\Delta\epsilon_1$ and $\Delta\epsilon_2$ in the probed spectral range. Indeed, in the UV, the real and imaginary part of the dielectric function have rather similar absolute values. For this reason, the optical reflectivity is equally sensitive to the reactive and the absorptive components of the dielectric function.

Hence, in order to calculate the pump-induced evolution of the ΔA from the $\Delta R/R$ data, we proceed as follows. We model the steady-state SE data using a set of Lorentz oscillators, we calculate the equilibrium reflectivity (R_0), and we fit the measured transient reflectance $R_{\mathrm{exp}}(t)/R_{\mathrm{exp}}$ with a differential model ($R(t)$ - R_0)/R_0, where $R(t)$ is a model for the perturbed reflectivity obtained by variation of the parameters used to fit the equilibrium data as a function of the pump-probe delay t. We adopt this approach to treat our $\Delta R/R$ data to avoid possible systematic errors that can be produced by the typical analysis through KK transformations. The SE spectra were fitted using a dielectric function of the form

$$\epsilon(\omega) = \epsilon_\infty + \sum_{\mathrm{j}} \frac{\omega_{\mathrm{pj}}^2}{\omega_{0\mathrm{i}}^2 - \omega^2 - i\Gamma_{\mathrm{j}}\omega} \tag{1}$$

where ϵ_∞ is the high-frequency dielectric constant, and ω_{pj}^2, $\omega_{0\mathrm{j}}^2$, Γ_{j} are, respectively, the plasma frequency, the transverse frequency and the linewidth (scattering rate) of the j-th Lorentz oscillator. The absorbance is then given by: $A(\omega) = \omega\ \mathrm{Im}\sqrt{\epsilon}$.

For the fitting of the transient data, the Lorentz oscillators in our experimental range are allowed to change in order to reproduce the dynamical reflectivity. This procedure enables to extract the transient dielectric function $\Delta\epsilon(\omega,t) = \Delta\epsilon_1(\omega,t) + i\Delta\epsilon_2(\omega,t)$ and finally leads to the evaluation of the ΔA for the single crystals.

Supplementary Note 4: *Ab initio* calculations - Frozen-lattice results

The calculated GW direct bandgap at the Γ point is 4.07 eV, at Z it is 4.13 eV, and the indirect bandgap (between Γ and a *k*-point close to X) is 3.61 eV. The bandgap at the middle point of the Γ-Z line is 3.96 eV. These values have been converged up to 5 meV, and the two codes used for the calculations give the same results, despite the use of a different plasmon pole models for the frequency integration in the GW method. The present fully-converged minimum GW quasiparticle correction amounts to 1.40 eV, which is smaller than the value of 1.69 eV from Ref. 2 (the difference comes from the smaller number of bands and *k*-points used in Ref. 2), highlighting the careful and exhaustive convergence evaluation done in the present work.

The symmetry-line along Γ-Z shows nearly parallel dispersion curves for the CB and VB edges. This peculiar shape of electronic states along Γ-Z plays a fundamental role in the optical properties of the material, as it dictates the nature and binding of the lowest excitons in anatase TiO_2 (see below). The nearly parallel dispersion observed in the theoretical bandgap allows us to use the direct gap at Γ as a very good approximation to estimate the bound direct nature of the exciton to be compared with the experimental data. Due to the band structure shape along the Γ-Z high-symmetry direction, we also underline that an accurate *k*-point sampling is especially critical for the quality of the optical spectra, since the main excitons are built up from optical transitions with contributions from a small region of the BZ. The CB and VB in this region display a wormlike shape aligned along the Γ-Z direction.

In Supplementary Fig. 10, we compare the results obtained with the GW implementations in BerkeleyGW and Yambo codes obtained at the same level of accuracy. Yambo calculations were performed using a 12×12×12 unshifted grid whereas a randomly shifted grid of 8×8×8 *k*-points was employed in the BerkeleyGW calculations. Thus, both approaches employ roughly 500 *k*-points. This shows the equivalence between the two implementations. To get the fully-converged optical spectra shown in the main text, a denser grid is required. Most importantly, the main effect of the stringent convergence obtained here with respect to *k*-points and number of bands is given by the shape of exciton I (see Supplementary Fig. 11). This charge excitation, split in two small peaks at low convergence[2,3] (or a main peak with a shoulder), becomes a unique, uniform peak, similar to the one observed in the experiment (dark red curve in Supplementary Fig. 11).

The fine k-sampling is needed, since the main optical transitions contributing to exciton I comes from the Γ-Z line, with bands almost parallel and flat. The two-dimensional exciton I for **E** ⊥ c (at 3.76 eV) has indeed a major contribution from the transition from the top of the VB to the bottom of the CB at the middle point in the Γ-Z line. To a lesser extent, significant contributions come from the k-points lying along the Γ-Z line and close to it in every direction. The contribution increases gradually when approaching the aforementioned Γ-Z middle point. Even if the GW direct electronic band gap of 3.96 eV, that is located at the middle point along the Γ-Z line, is used as a reference energy to calculate the exciton binding energy (E_{B}), the bound nature of exciton I is still confirmed (with $E_{\mathrm{B}} = 50$ meV). A phenomenological Lorentzian broadening of 0.12 eV was applied to reproduce the experimental spectra (see Supplementary Fig. 12 for a comparison of the measured spectrum with the bare BSE eigenvalues, showing that mainly one eigenvalue is contributing to the exciton peak I and that the measured lifetime is not of electronic origin but is due to the strong electron-phonon coupling in this material).

The energy, shape and reciprocal space contributions for peak II highlights its bulk-resonance character, most evident as its offset coincides with the RPA-GW absorption rise. On the other hand, we found that exciton III for **E** ∥ c (at 4.28 eV) is of a more complex nature, with a mixed contribution of bound excitons and delocalized resonant transitions. The former have the dominant character and the contributing transitions are found throughout the BZ, while the main contribution for the latter is concentrated in few points close to the Γ-Z line in the region around Z. The energy and shape of exciton III confirm the analysis of a mixed bulk resonance and localized character, as the continuum onset in RPA-GW appears to undergo an intensity enhancement. The mixed bulk-localized nature makes it less straightforward to estimate its E_{B}. Assuming the RPA-GW onset at 4.40 eV for **E** ∥ c as the reference energy for evaluating E_{B}, we estimate $E_{\mathrm{B}} \sim$150 meV.

The slight shift of the calculated exciton III with respect to our experimental value (0.1 eV) is also investigated in detail. Increasing the number of k-points and bands does not allow to obtain a match to experiment as good as for exciton I. We can also exclude possible effects of anisotropic screening, as increasing the parameters of the local field effects and separating the screening along the a- and c-axis components do not lead to significant changes in the

spectrum. The peak maximum seems instead to be related, in a nonlinear manner, to the lattice constants. The a-axis lattice constant from *ab initio* optimization is in excellent agreement with experimental data (3.79 Å vs 3.78 Å), while the c-axis lattice constant is slightly (1%) overestimated. However, when using the experimental lattice parameters, the position of peak III is blueshifted by 0.20 eV from that obtained with the PBE parameters, thus worsening the agreement with experiment.

Finally, the presence of dark excitons in anatase TiO_2 at energies below the bright exciton I at 3.76 eV is ruled out by our spin-resolved optical BSE calculations. Indeed, the calculated lowest exciton in anatase TiO_2 is a singlet and it is optically active. This does not apply to the rutile phase of TiO_2,[2] where the lowest exciton is an optically dark triplet state.

Supplementary Note 5: *Ab initio* calculations - Comparison with previous studies

The computational data presented in this paper are novel and conclusive in many respects, despite being performed at the same level of theory (DFT + GW + BSE) as in some previous reports.[2–5] First of all, this is the first available comparison between the theoretical and the experimental electronic gap for pristine and doped anatase TiO_2. As the experimental gap at the Γ point of the BZ is measured here for the first time, in the past there has been a high degree of uncertainty concerning the computational results. The reported direct electronic gaps at Γ ranged from 4.14 eV (PBE-G_0W_0)[3] to 3.78 eV (PBE-G_0W_0)[4] and 4.29 eV (PBE-G_0W_0).[2] The use of methods beyond GGA-G_0W_0 provided even larger values (4.05 eV, G_0W_0 on top of hybrid functional HSE06 for the indirect gap,[4] larger than our 3.61 eV; 5.28 eV, self-consistent GW different flavours[10]).

Moreover, the GW calculations presented here go beyond the ones reported in previous works, as they address the combined effects of electron doping, temperature-induced lattice expansion and electron-phonon coupling on the direct gap at the Γ point of the BZ. Such investigations rule out the role of the BGR in the description of the quasiparticle gap and enable the identification of electron-phonon coupling as the main source of renormalization of the quasiparticle gap.

A second important point concerns the comparison of the refined theoretical data here reported

with SE data. Indeed, the previous zero-temperature computational calculations relied on a comparison with the dielectric function at 100 K of Ref. 1, which was extracted via a KK analysis from normal-incidence reflectivity data. We note again that the two different energy scales used in Ref. 1 to display the data of the dielectric function for $\mathbf{E} \perp c$ and $\mathbf{E} \parallel c$ has led to an error of digitalization by early computational works, which has propagated in the literature[2–5] on anatase TiO_2. Here, we correctly compare our new calculations with the low-temperature dielectric function of the material, measured directly via SE and not extracted through a KK analysis. In this way, we clarify the precise peak positions and shape in the optical absorption for the material.

Besides the higher convergence achieved in comparison with previous works, our evaluation of the optical spectra presents novel results, since we included doping, electron-phonon coupling and the role of indirect transitions, and discussed their effects on the energy and shape of the optical peaks (see the following sections).

Supplementary Note 6: *Ab initio* calculations - Doped anatase TiO_2

Within the same theoretical framework used for pristine anatase TiO_2, we perform calculations for the case of uniformly doped anatase TiO_2, to verify computationally that the influence of doping on both the electronic gap and optical response can be disregarded. Here, we show the results for two cases of uniform excess electron density $n = 10^{19}$ cm^{-3} and $n = 10^{20}$ cm^{-3}. The calculated GW gap (both direct and indirect gap) are similar to the pristine anatase TiO_2 case, with an increase of 1 meV for the doping of $n = 10^{19}$ cm^{-3} and of 17 meV for $n = 10^{20}$ cm^{-3}. These results complement the experimental ARPES data, demonstrating that the electronic gap from doped samples is a very good value to describe also the gap of pristine anatase TiO_2. In the presence of doping, two competing effects contribute to changing the electronic gap of a semiconductor, with either a redshift or blueshift depending on which effect dominates. The CB filling is responsible for the blueshift, while the change in the long-range Coulomb screening (becoming slightly metallic) is responsible for the redshift. In anatase TiO_2, the dominant effect for the considered doping ranges is the CB filling. For the doping values relevant in our measurements, the correction is well below the computational error, therefore, there is no

detectable effect of doping in the electronic band gap. This is supported by the calculation of the optical response, as the position and shape of peak I in ϵ_{2a} (Supplementary Fig. 13) do not change for $n = 10^{19}$ cm^{-3}. These results justify the strategy, followed in the main text, to experimentally estimate the exciton E_B from the ARPES measurements in the doped samples.

Supplementary Note 7: *Ab initio* calculations - Electron-phonon and temperature effects

Our calculations taking into account the role of the electron-phonon coupling reveal a GW bandgap increase of 60 meV (in the case of the E_u mode) and 80 meV (in the case of the A_{2u} mode) at 300 K, compared to the zero temperature value. We correct this value by also considering the lattice expansion effect. By using the thermal expansion coefficient in Ref. 12, we determine that the a and c lattice parameters of anatase TiO_2 increase in 0.1 % and 0.3 %, respectively, from 0 to 300 K. The inclusion of both the phonon-induced and thermal expansion-induced effects leads to a net blueshift of the bandgap of about 30 meV (in the case of the E_u mode) and 50 meV (in the case of the A_{2u} mode) from zero temperature to 300 K. A similar trend was recently reported for rutile TiO_2, where the electronic gap (evaluated within the thermal lines method for electron-phonon coupling) has a non-monotonic behavior with temperature.[11] Additionally, we solve the BSE on top of the temperature-corrected GW and find a net blueshift of roughly 80 meV (in the case of the E_u mode) 70 meV (in the case of the A_{2u} mode) at 300 K, which is in line with our SE measurements (blueshift of 40 meV from 20 K to 300 K).

Supplementary Note 8: *Ab initio* calculations - Direct exciton in an indirect band gap material

We consider a supercell composed of 3×3×2 conventional unit cells (12 atoms) which leads to a total of 216 atoms in the supercell. This implies the inclusion of 648 phonons. Although the employed number of phonons is still limited, it provides a first approximation of the effect of the indirect gap in the renormalization of the excitonic peak. We perform two molecular

dynamics simulation runs at temperatures of 20 K and 300 K. The molecular dynamics runs were carried out using a Nosé-Hoover chain thermostat. A total of 5 snapshots were randomly chosen in the interval between 5 ps and 10 ps of the run for each temperature. To investigate if the position of the excitonic peak changes when accounting for the indirect nature of the material, we perform similar calculations for the primitive unit cell of anatase TiO_2 at the same level of theory and convergence. We obtain a negligible blueshift of 30 meV, which indicates that the indirect bandgap nature of anatase TiO_2 does not play a significant role in the exciton properties, beyond adding an Urbach tail at the lower energy side of the peak.

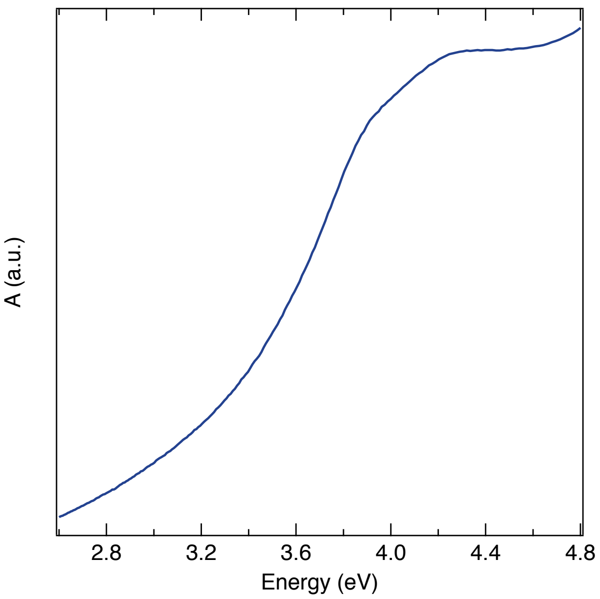

Supplementary Figure 1: Steady-state absorption spectrum of anatase TiO_2 NPs. Room temperature steady-state absorption spectrum of anatase TiO_2 NPs dispersed in aqueous solution.

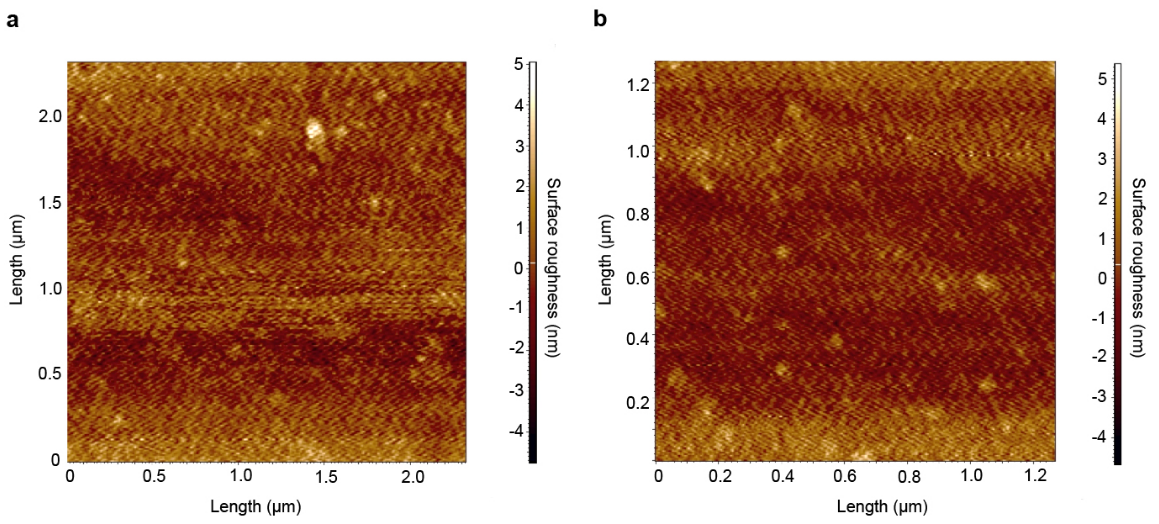

Supplementary Figure 2: Atomic Force Microscopy images of anatase TiO_2 single crystals.
(a, b) Roughness characterization of the (010)-oriented polished surface of the reduced anatase TiO_2 single crystal used for the SE measurement. The images are taken using Atomic Force Microscopy and the average surface roughness is estimated around 0.9 nm.

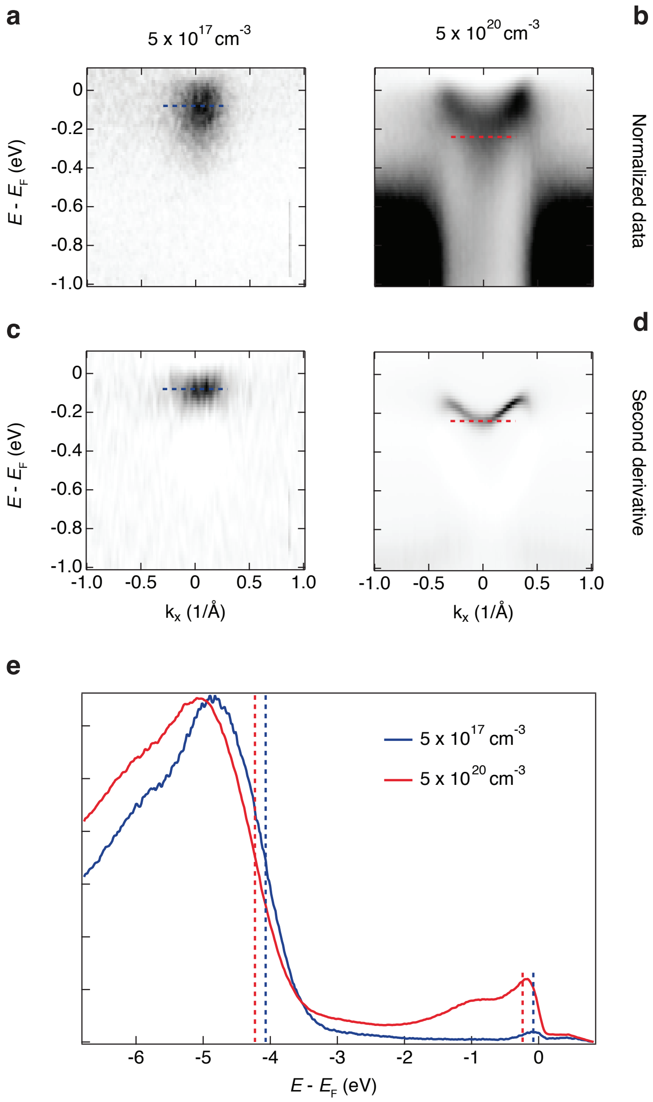

Supplementary Figure 3: Doping dependence of the ARPES data.
(a,b) Energy vs. momentum dispersion of the bottom of the CB for n-doped samples with $n = 5 \times 10^{17}$ cm^{-3} and $n = 5 \times 10^{20}$ cm^{-3}. **(c,d)** Second derivative maps of the energy vs. momentum dispersion obtained from panels **(a,b)**, respectively. **(e)** Energy distribution curves at the Γ point of the BZ for the two considered doping levels. The dashed vertical lines identify the positions of the quasiparticle energies for the VB and CB in the two different samples. The spectra are referenced to E_{F}.

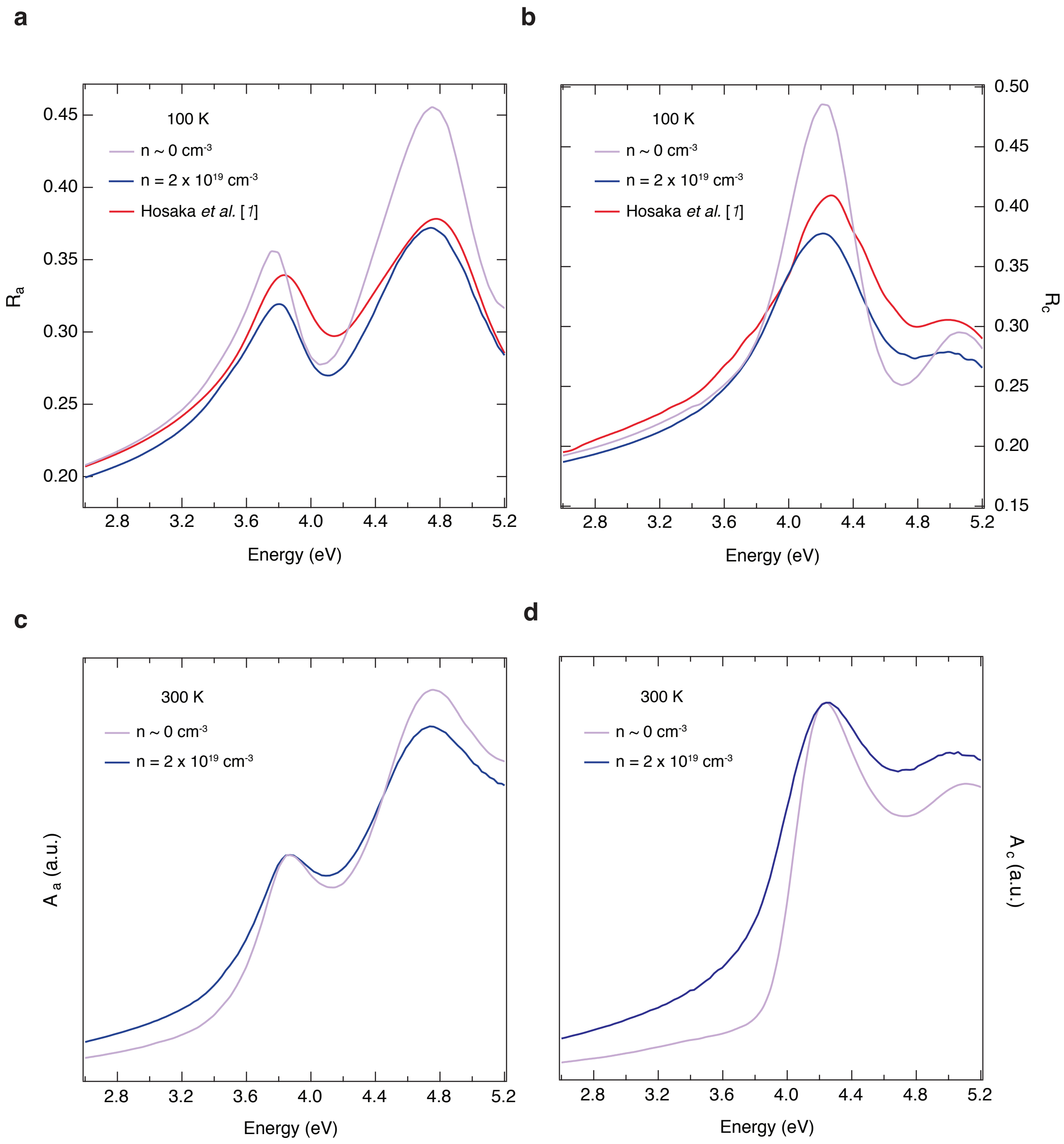

Supplementary Figure 4: Steady-state reflectance and absorption of anatase TiO_2 single crystals.
(a,b) Steady-state reflectivity spectra of (010)-oriented anatase TiO_2 single crystals at 100 K. The electric field is polarized along: **a** the a-axis; **b** the c-axis of the crystals. The data derived from our SE measurements are depicted in violet for the pristine ($n \sim 0$ cm^{-3}) crystal and in blue for the n-doped ($n = 2 \times 10^{19}$ cm^{-3}) crystal, while the reflectivity measured in Ref. 1 is reported in red lines. **(c,d)**, Normalized steady-state absorption spectra of (010)-oriented anatase TiO_2 single crystals at 300 K. The electric field is polarized along: **c** the a-axis; **d** the c-axis. The data derived from our SE measurements are depicted in violet for the pristine ($n \sim 0$ cm^{-3}) crystal and in blue for the n-doped ($n = 2 \times 10^{19}$ cm^{-3}) crystal.

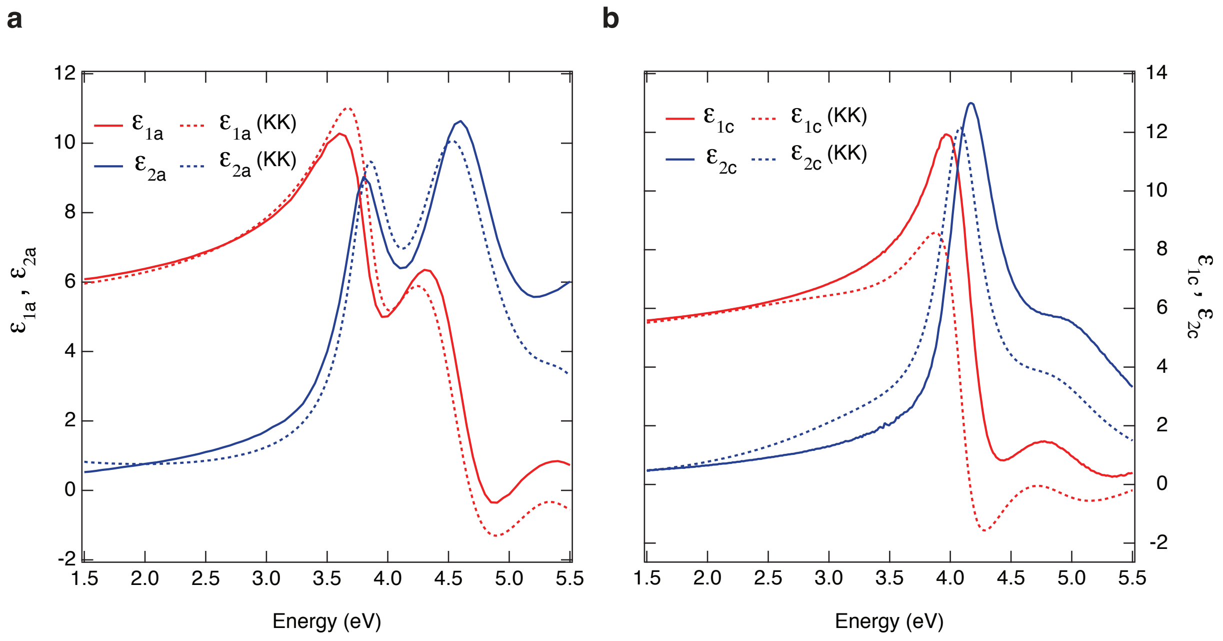

Supplementary Figure 5: Complex dielectric function of anatase TiO_2 single crystals at RT.

The electric field is polarized along **(a)** the a-axis and **(b)** the c-axis. The real part, $\epsilon_1(\omega)$, is plotted in red, while the imaginary part, $\epsilon_2(\omega)$, in blue. The solid-line curves depict the data directly extracted from SE, while the dashed lines are calculated by a KK analysis of reflectivity.

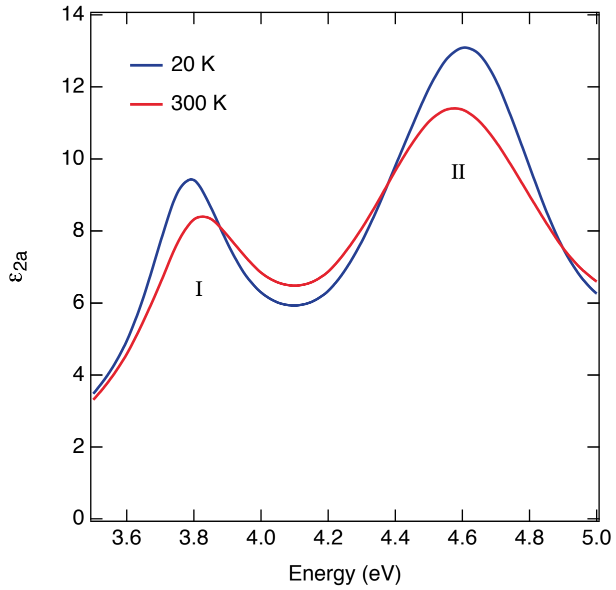

Supplementary Figure 6: Temperature dependence of exciton peak I.
Imaginary part of the dielectric function at 20 K (blue curve) and 300 K (red curve) for $\mathbf{E} \perp$ c. The exciton I is observed to undergo an anomalous blueshift of its peak energy, while the charge excitation II displays a conventional redshift.

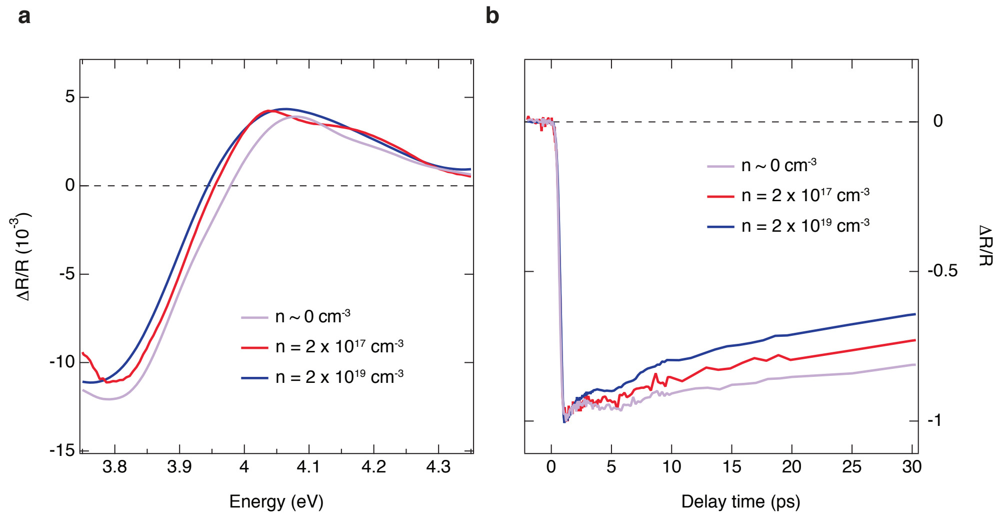

Supplementary Figure 7: Ultrafast broadband UV reflectivity on different classes of anatase TiO_2 single crystals at RT.
The doping levels are indicated in the labels. Both pump and probe polarizations lie along the a-axis and the pump photon energy is set at 4.10 eV: **(a)** Transient spectrum at the fixed time delay of 6 ps. **(b)** Normalized temporal traces at a fixed probe energy of 3.82 eV.

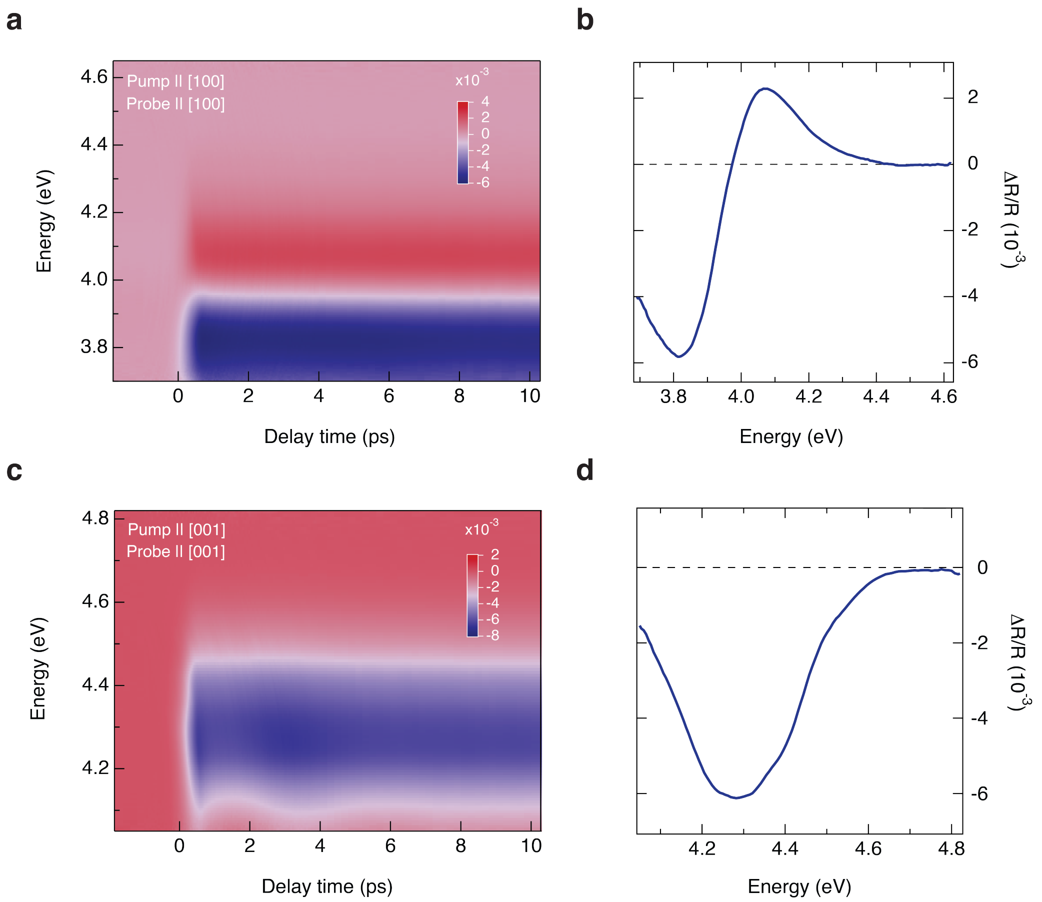

Supplementary Figure 8: Ultrafast anisotropic response of anatase TiO_2 single crystals.
Ultrafast broadband UV reflectivity on a (010)-oriented anatase TiO_2 single crystal ($n = 2 \times 10^{19}$ cm^{-3}) at RT. **(a,b)** Colour-coded maps of $\Delta R/R$ measured upon photoexcitation at 4.40 eV. **(c,d)** Transient spectra, obtained from a cut at 1 ps in the experimental conditions reported for **a** and **b**, respectively.

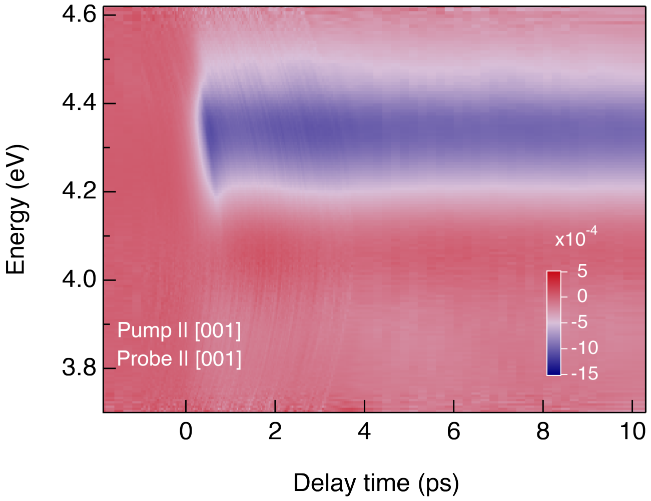

Supplementary Figure 9: Absence of low-energy spectral features in the c-axis ultrafast response.
Colour-coded map of $\Delta R/R$ from a (010)-oriented single crystal ($n = 2 \times 10^{19}$ cm^{-3}) measured at RT upon photoexcitation at 4.40 eV and with pump and probe beams polarized along the c-axis. The probe photon energy covers the spectral range 3.70 - 4.60 eV, which demonstrates the absence of emerging features at low energies.

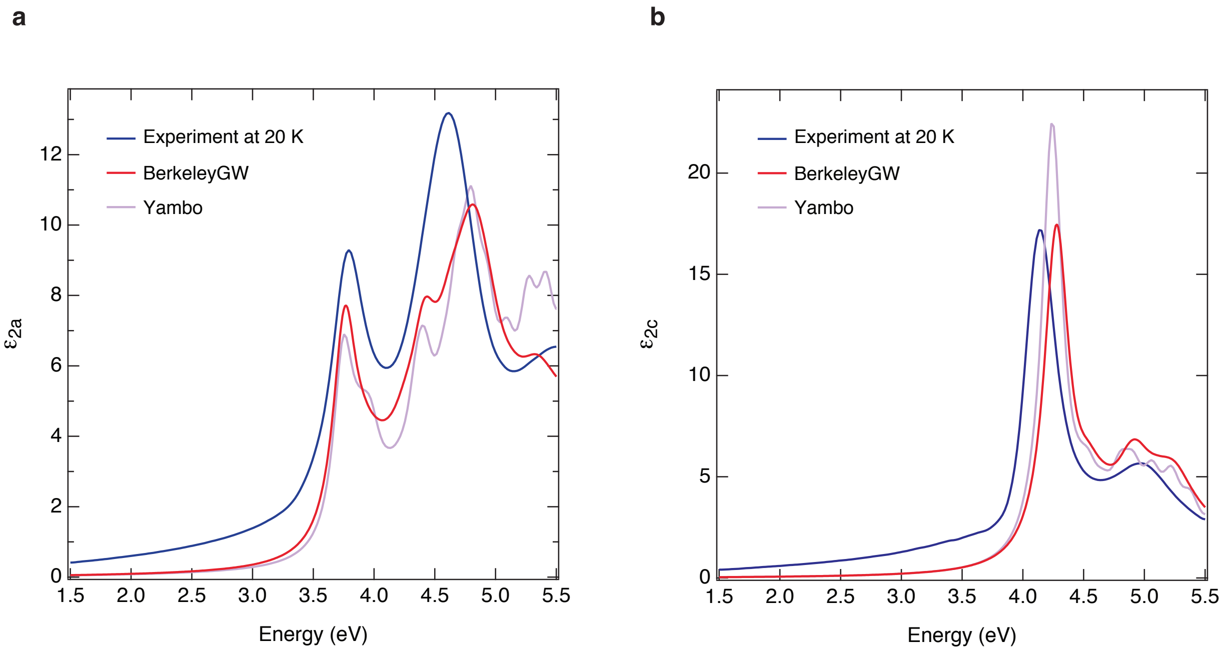

Supplementary Figure 10: Comparison between the experimental SE data and BSE calculations.
Both BerkeleyGW (red curve) and Yambo (violet curve) data are evaluated using the highest convergence parameter values described in the text. Experimental data at 20 K are shown in blue. For BerkeleyGW, they correspond to the best converged spectra (both for peaks shape and position). In Yambo, the spectra have been obtained with a less dense k-grid, leading the a-axis spectrum to show a spurious shoulder above exciton peak I, as in the previously published works.[2] The fully-converged spectra (red curves) show a single peak in agreement with the experimental data. For light polarized along the a-axis, the agreement between the two calculations is excellent.

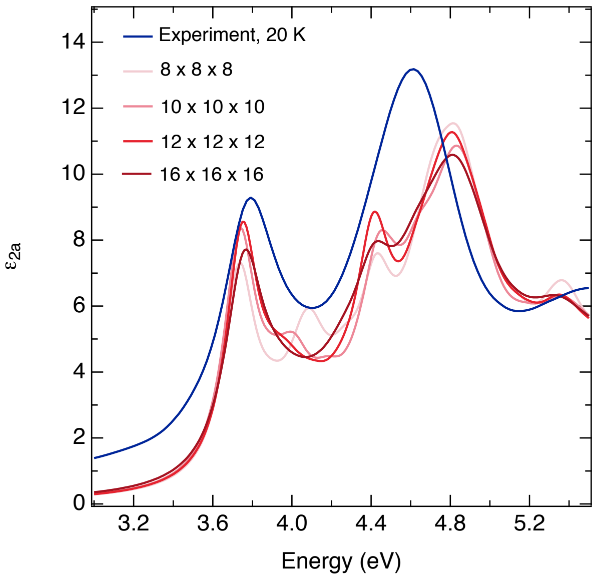

Supplementary Figure 11: Convergence test for the dielectric function. Convergence test performed with respect to the size of the k-points grid using the BerkeleyGW code, in red-colour scale. For comparison, the experimental data at 20 K are also shown (blue curve). To obtain the proper shape of the spectra, it is necessary to use a very large k-point grid together with a very large number of bands as described in the text.

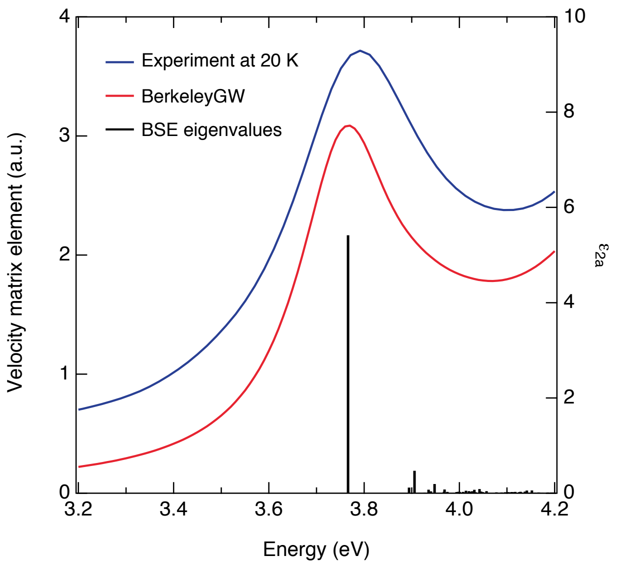

Supplementary Figure 12: Eigenvalue analysis on exciton peak I.
Black bars represent the square of the transition matrix elements of the velocity operator along the a-axis ($|\mathbf{E}_\mathrm{a} \cdot \langle 0|\ \mathbf{v}\ |\mathrm{S}\rangle\ |^2$), corresponding to exciton states S contributing to the peak I. This quantity is related to the oscillator strength f_S by $f_\mathrm{S} = (2\ |\ \mathbf{E}_\mathrm{a} \cdot \langle 0|\ \mathbf{v}\ |S\rangle\ |^2)/E_\mathrm{S}$, where E_S is the excitation energy corresponding to exciton state S. The experimental $\epsilon_{2\mathrm{a}}$ (blue curve) and the full-converged BerkeleyGW calculations with a phenomenological Lorentzian broadening of 0.12 eV (red curve) are also compared.

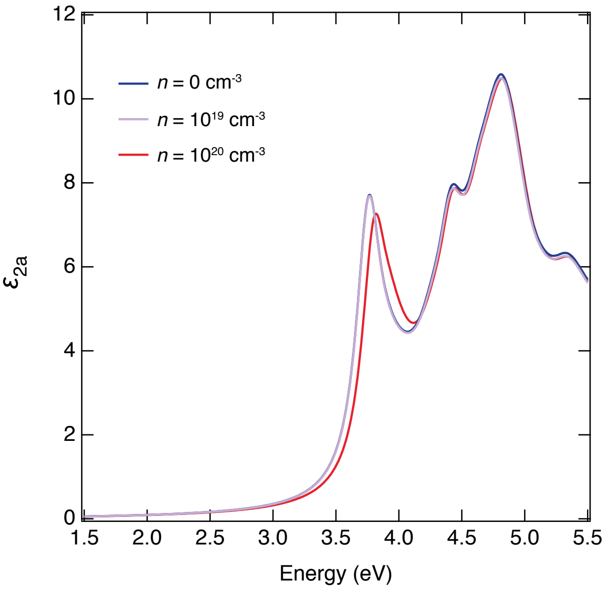

Supplementary Figure 13: Doping dependence of the BSE spectrum. Comparison between the calculated ϵ_{2a} for pristine and n-doped anatase TiO_2. The optical response of the n-doped TiO_2 with $n = 10^{19}$ cm^{-3} (violet curve) overlaps almost completely to the pristine case (blue curve), showing that this doping level does not produce a significant effect the peak energy of feature I. Only when n is increased to 10^{20} cm^{-3} (red curve), the peak energy of feature I is found to blueshift of $\sim$ 50 meV

Supplementary References

[1] Hosaka, N., Sekiya, T., Satoko, C. & Kurita, S. Optical properties of single-crystal anatase TiO_2. *J. Phys. Soc. Jap.* **66**, 877-880 (1997).

[2] Chiodo, L. *et al.* Self-energy and excitonic effects in the electronic and optical properties of TiO_2 crystalline phases. *Phys. Rev. B* **82**, 045207 (2010).

[3] Kang, W. & Hybertsen, M. S. Quasiparticle and optical properties of rutile and anatase TiO_2. *Phys. Rev. B* **82**, 085203 (2010).

[4] Landmann, M., Rauls, E. & Schmidt, W. G. The electronic structure and optical response of rutile, anatase and brookite TiO_2. *J. Phys. Condens. Matter* **24**, 195503 (2012).

[5] Lawler, H. M. *et al.* Optical to UV spectra and birefringence of SiO_2 and TiO_2: First-principles calculations with excitonic effects. *Phys. Rev. B* **78**, 205108 (2008).

[6] Reynolds, D. C., Look, D. C. & Jogai, B. Combined effects of screening and band gap renormalization on the energy of optical transitions in ZnO and GaN. *J. Appl. Phys.* **88**, 10, 5760-5763 (2000).

[7] Wegscheider, W. *et al.* Lasing from excitons in quantum wires. *Phys. Rev. Lett.* **71**, 4071 (1993).

[8] Ambigapathy, R. *et al.* Coulomb correlation and band gap renormalization at high carrier densities in quantum wires. *Phys. Rev. Lett.* **78**, 3579 (1997).

[9] Das Sarma, S. & Wang, D. W. Many-body renormalization of semiconductor quantum wire excitons: Absorption, gain, binding, unbinding, and Mott transition. *Phys. Rev. Lett.* **84**, 2010 (2000).

[10] Kang, G., Kang, Y. & Han, S. Influence of wave-function updates in GW calculations on titanates. *Phys. Rev. B* **91**, 155141 (2015).

[11] Monserrat, B. Correlation effects on electron-phonon coupling in semiconductors: Many-body theory along thermal lines. *Phys. Rev. B* **93**, 100301(R) (2016).

[12] Rao, K. V. K., Naidu, S. V. N. & Iyengar, L. Thermal expansion of rutile and anatase. *J. Am. Cer. Soc.* **53**, 124–126 (1970).
