# Supplementary material for: Strongly bound excitons in anatase TiO2 single crystals and nanoparticles
Source: Nat Commun. 2017 Apr 13;8:13. doi: 10.1038/s41467-017-00016-6 (PMC5432032; doi:10.1038/s41467-017-00016-6)
Supplement: Supplementary file 2 — Reviewer reports and authors' response from the peer review of this Article at Nature Communications [file 41467_2017_16_MOESM2_ESM.pdf]

Editorial Note: this manuscript has been previously reviewed at another journal that is not operating a transparent peer review scheme. This document only contains reviewer comments and rebuttal letters for versions considered at Nature Communications.

Reviewers' comments:

Reviewer #4 (Remarks to the Author):

The authors report a combined theoretical (DFT+GW+BSE) and experimental (ARPES+SE) study on the electronic structure and optical response of anatase TiO2. This material has been extensively investigated before. Still, the authors add significantly to our understanding of its optical absorption onset: They show that it is dominated by an exciton that is essentially confined to a single atomic plane.

This finding is new, and - at least to me - unexpected. I am not sure, however, if this finding is in fact relevant for any application of titanium dioxide. Moreover, and this seems to me even more important, the authors fail to explain why a two-dimensional exciton forms in this specific material. Which structural, chemical or electronic peculiarities cause this very special character?

On the method point of view: The authors stress that they present "conclusive" computational data. This may or may not be the case. Certainly they go in some aspects beyond previous calculations on TiO2. On the other hand, it has to be said that for a variety of materials more advanced calculations have been published recently. This concerns, e.g., a self-consistent solution of the Dyson equation or the full inclusion of the phonon dispersion relation in the electronic structure calculation. I do not believe that improving the calculations along these directions will provide any new physical insight. Still, the authors claim to be "conclusive" seems out of place.

Concerning the publication criteria: There is no doubt concerning the work being technically sound and the results being novel and well founded. About the importance I am not really sure. Possibly a more specialized journal would be more appropriate.

Reviewer #5 (Remarks to the Author):

The present study examines physical properties of the excitons in anatase TiO₂ by both experimental and theoretical approaches. Although many studies have been devoted to elucidate the optical response of TiO₂ (both rutile and anatase forms), experimental verification of the excitons has been insufficient. The present experimental and theoretical study ambiguously indicates the formation of the bound excitons in anatase TiO₂. One of noteworthy achievements is the experimental determination of the exciton binding energy (180 meV) by a sophisticated way. The technique demonstrated in this study may be useful to experimentally verify the exciton binding energy of indirect band gap materials, although certain conditions must be cleared (for example, it cannot be applied for rutile TiO₂ because doped electrons do not fill the conduction band). As far as I know, this is the first claim to experimentally verify the exciton binding energy of anatase TiO₂. The present study also reveals that the exciton properties are essentially the same between the bulk single crystal and the nanoparticles (with the size of 25 nm) suspended in the solution.
Although the above mentioned experimental findings are important, the theoretically-derived conclusions, i.e. the unique 2D spatial distribution of the exciton in the 3D material as well as the

intermediate nature between Wannier-Mott and Frenkel excitons, are not newly introduced concept. These exciton features have already been proposed by the authors' preceding studies (Refs. 10 and 37). Especially, very similar discussions on the imaginary part of the dielectric constant (BSE-GW versus RPA-GW) and on the exciton distribution have already been done in Ref. 10. Although higher precision computations were carried out in the present study than those in the preceding studies, this fact severely deteriorates the novelty of the study. Thus, the authors' strong claim on this point sounds odd to me.
Another drawback of this manuscript is that, although the determined exciton binding energy is unexpectedly large (much larger than the exciton in rutile TiO₂), the authors did not make any effort to elucidate how this large binding energy is emerged and how the spatial extension affects the binding energy. If these discussions are included, the manuscript will be much improved.
In conclusion, the paper is not suitable for publication in Nat. Commun.

Following three points are my recommendation:

(1) The claim that the lowest exciton has a unique 2D distribution must be weakened, since it is not the original claim of the present study.
(2) The origin and the mechanism of the large exciton binding energy of anatase TiO₂ should be discussed while comparing binding energies of other oxide materials.
(3) If there is a correlation between the spatial distribution and the exciton binding energy, it must be discussed.

Other recommendations, questions and comments are given below:

(4) In the resent study, the n-doped anatase crystal was prepared by annealing at 700°C in CO atmosphere. Thus, the excess electrons should be offered by bulk O vacancies. On the other hand, it is known that UV irradiation induces O vacancies in the surface region to form 2DEG [PRB 92, 041106(R) (2015)]. Is there any possibility that the observed metallic band originates from the 2DEG? If so, the bottom of the metallic band do not coincide with the CBM.
This concern arises because, in the present study, the crystal was annealed in 35 mbar of O₂ for 30 min. This treatment should result in the compensation of O vacancies in the surface and subsurface regions. So the metallic peak must not be observed if the vacancy free surface was prepared. If the UV irradiation reads to the surface O vacancies and a metallic band appears, it is possible to interpret that the 2DEG gives the band. The photo energy used in the present study was 128 eV, which is a very surface sensitive condition. Therefore, the authors must guarantee that the observed metallic band is the bulk CB.

(5) How was the excess electron density determined? Transport measurements or ARPES?

(6) "ii) a reduction of the oscillator strength of all peaks, ..." (p. 8)
The peaks II and III are well above the Fermi level. Therefore, the reduction cannot be ascribed to the spectral weight transfer.

(7) "The insensitivity of peak I for different doping levels" (p. 11)
"The insensitivity" of what? Energy or intensity?

(8) "As far as peak I is concerned, it extends two-dimensionally on the (001) plane with a radius of 15 angstrom, being almost confined to a single atomic plane along the c-axis." (p. 12)
From Fig. 3a, the radius of the exciton distribution is much larger than 1.5 nm. From Fig. 3d, the distribution seems not to be confined in a single plane but in two planes. Not "along the c-axis", but "in the ab-plane".

(9) The binding energy of exciton III is evaluated to be 150 meV (p. 13).

How was this value deduced?

(10) Since Fig. 4b shows absorption spectra of only one doped (or pristine) TiO₂ sample, "anatase TiO₂ single crystal (with varying degrees of oxygen vacancies)" (p. 15) is misleading.

(11) "Both features have a relatively narrow width of 250 meV." (p. 15)
I see the peak is much broader than 0.25 eV (Fig. 4).

(12) "The reduced (pristine) form of anatase TiO₂" (p. 18) may lead confusion. "Pristine crystal" and "n-doped crystal" should be clearly defined with the excess electron densities. The density of the Cu-doped crystal is also desired.

(13) The photon energy used for the ARPES measurements must be indicated in the main text, though it is offered in the supplemental. The energy resolution of the system is also desired.

(14) Typos:
p. 10 ; (Fig. 2a-b, red line) → (Fig. 2c-d, red line)
p. 20 ; 5% fluoridic acid → 5% fluoric acid or 5% hydrofluoric acid

Reviewer #4 (Remarks to the Author):

The authors report a combined theoretical (DFT+GW+BSE) and experimental (ARPES+SE) study on the electronic structure and optical response of anatase TiO_2. This material has been extensively investigated before. Still, the authors add significantly to our understanding of its optical absorption onset: They show that it is dominated by an exciton that is essentially confined to a single atomic plane.

We thank the referee for her/his positive appreciation of our work.

This finding is new, and - at least to me - unexpected. I am not sure, however, if this finding is in fact relevant for any application of titanium dioxide. Moreover, and this seems to me even more important, the authors fail to explain why a two-dimensional exciton forms in this specific material. Which structural, chemical or electronic peculiarities cause this very special character?

The relevance of our findings with regard to application is beyond the scope and aims of our paper. We have added a discussion at the end of the new version, which compares our results with those on other titanates. This comparison allows a rationalization as to why anatase TiO_2 sustains a 2D exciton in contrast to the other titanium oxides.

On the method point of view: The authors stress that they present "conclusive" computational data. This may or may not be the case.

Certainly they go in some aspects beyond previous calculations on TiO_2. On the other hand, it has to be said that for a variety of materials more advanced calculations have been published recently. This concerns, e.g., a self-consistent solution of the Dyson equation or the full inclusion of the phonon dispersion relation in the electronic structure calculation. I do not believe that improving the calculations along these directions will provide any new physical insight. Still, the authors claim to be "conclusive" seems out of place.

As the referee notes, our calculations go beyond the previous state-of-the-art calculations reported for this material and other titanates, because we included the finite doping effect, the electron-phonon and temperature effects as well as the treatment of indirect transitions. Concerning the conclusiveness of our calculations, we have inadequately expressed ourselves; as we meant that all those new ingredients are required to they fully wrap up the experimental results into a coherent and consistent interpretation. This was lacking until now. We made this clear in the new manuscript. The novelty of our study actually lies in the experimental demonstration of the existence of the excitons both under equilibrium and nonequilibrium conditions in different classes of systems (single crystals and nanoparticles used in the applications). Following the comment by the referee, we rephrased our paper in terms of novelty, highlighting how our experimental study goes beyond the current knowledge on this extensively studied material.

Concerning the publication criteria: There is no doubt concerning the work being technically sound and the results being novel and well founded. About the importance I am not really sure. Possibly a more specialized journal would be more appropriate.

Again, because of the huge popularity of the studied system in light-driven applications, and because our work provides new insights into the physics of materials whose basic physical properties were supposed to be "well characterized", we believe that a more specialized journal will not be appropriate. Furthermore, the obtained results were possible through a unique combination (as actually also appreciated by reviewer 5) of different methods (both experimental and theoretical), which are state-of-the-art. In particular, we presented the first demonstration of ultrafast two-dimensional UV spectroscopy in condensed matter systems. For these reasons, we believe that our paper has the potential to be received by a wide and diverse audience as that of Nature Communications.

Reviewer #5 (Remarks to the Author):

The present study examines physical properties of the excitons in anatase TiO_2 by both experimental and theoretical approaches. Although many studies have been devoted to elucidate the optical response of TiO_2 (both rutile and anatase forms), experimental verification of the excitons has been insufficient. The present experimental and theoretical study ambiguously indicates the formation of the bound excitons in anatase TiO_2. One of noteworthy achievements is the experimental determination of the

exciton binding energy (180 meV) by a sophisticated way. The technique demonstrated in this study may be useful to experimentally verify the exciton binding energy of indirect band gap materials, although certain conditions must be cleared (for example, it cannot be applied for rutile TiO_2 because doped electrons do not fill the conduction band). As far as I know, this is the first claim to experimentally verify the exciton binding energy of anatase TiO_2. The present study also reveals that the exciton properties are essentially the same between the bulk single crystal and the nanoparticles (with the size of 25 nm) suspended in the solution.

We thank the referee for her/his appreciation of our experimental methodology and for recognising its novelty.

Although the above mentioned experimental findings are important, the theoretically-derived conclusions, i.e. the unique 2D spatial distribution of the exciton in the 3D material as well as the intermediate nature between Wannier-Mott and Frenkel excitons, are not newly introduced concept. These exciton features have already been proposed by the authors' preceding studies (Refs. 10 and 37). Especially, very similar discussions on the imaginary part of the dielectric constant (BSE-GW versus RPA-GW) and on the exciton distribution have already been done in Ref. 10. Although higher precision computations were carried out in the present study than those in the preceding studies, this fact severely deteriorates the novelty of the study. Thus, the authors' strong claim on this point sounds odd to me.

This comment is similar to that raised by reviewer 4 and we agree that the novelty of the theory was over emphasized. However, the main findings of this work come from the experimental side, namely the first demonstration of a strongly bound exciton in anatase TiO_2 single-crystals and nanoparticles and the observation of the exciton stability even at room temperature. In this respect we stress once more the novelty (and the need) of combining several state-of-the-art methods to come to our conclusions. Concerning the theory, we agree that the 2D nature of the exciton is not a new concept, as it was treated in Refs. 10 and 37 of the previous version of our manuscript. However, the present calculations include effects that had hitherto not been considered before for this material: doping, electron-phonon coupling, temperature and treatment of indirect transitions. These calculations fully wrap up the experimental interpretation and the experiment-theory ensemble is, in our opinion, put on a very solid basis. Moreover we show that this very good agreement between experiments and theory was only possible with highly converged calculations that were beyond reach some years back.

Another drawback of this manuscript is that, although the determined exciton binding energy is unexpectedly large (much larger than the exciton in rutile TiO_2), the authors did not make any effort to elucidate how this large binding energy is emerged and how the spatial extension affects the binding energy. If these discussions are included, the manuscript will be much improved.

Again this is similar to reviewer 4's remark above and we refer to our answer. The discussion at the end of the paper goes into the details.

In conclusion, the paper is not suitable for publication in Nat. Commun.

We disagree with the reviewer as thanks to her/his comments, we now have a solid interpretation of this much-studied system.

Following three points are my recommendation:

(1) The claim that the lowest exciton has a unique 2D distribution must be weakened, since it is not the original claim of the present study.

We agree with the referee on this point, we focused our message on the experimental demonstration of the existence of bound excitons in anatase TiO_2 single-crystals and nanoparticles, and we softened our claim concerning the 2D exciton wavefunction. In this respect, even the title has been modified.

(2) The origin and the mechanism of the large exciton binding energy of anatase TiO_2 should be discussed while comparing binding energies of other oxide materials.

This has been dealt in our reply to reviewer 4. We also added a detailed discussion of the mechanism through which large binding energies emerge

in anatase TiO_2 via a comparison with the other important polymorph of TiO_2, i.e. rutile, and with the titanates having a perovskite-like structure.

(3) If there is a correlation between the spatial distribution and the exciton binding energy, it must be discussed.

We thank the referee for this comment. We included a detailed discussion concerning this aspect at the end of the paper, which adds value to our work. It is true that a localized exciton has a strong binding energy (like a Frenkel exciton) but the opposite does not hold: a clear example is the strongly bound exciton in $SrTiO_3$ (220 meV of binding energy, *i.e.* larger than in anatase TiO_2), which is completely delocalized in the system.

The exciton is a collective excitation of the electronic system and, as such, it involves many states of the electronic band structure. A necessary condition for this many-state transition to occur and for the exciton to form is that the electron and hole group velocities be nearly the same, *i.e.* that the gradients of the lowest CB and the highest VB are identical in a specific portion of the Brillouin zone. As a consequence, while the excitation energy of a single-particle transition is just the difference between the initial and final single-particle states, the excitation energy of an excitonic transition is lower than the fundamental band gap. The stabilization energy with respect to the uncorrelated particle-hole limit is the fingerprint for the collective nature of the state created in the absorption process. Thus, this quantity depends on the number of states taking part to the excitonic transition. So the exciton binding energy

depends on the details of the electronic structure and the nature of the screening (which has to be considered momentum- and energy-dependent). The degree of excitonic delocalization is instead influenced by the crystal structure in terms of packing of the polyhedra containing the atoms involved in the excitonic transitions.

Other recommendations, questions and comments are given below:

(4) In the present study, the n-doped anatase crystal was prepared by annealing at 700°C in CO atmosphere. Thus, the excess electrons should be offered by bulk O vacancies. On the other hand, it is known that UV irradiation induces O vacancies in the surface region to form 2DEG [PRB 92, 041106(R) (2015)]. Is there any possibility that the observed metallic band originates from the 2DEG? If so, the bottom of the metallic band do not coincide with the CBM. This concern arises because, in the present study, the crystal was annealed in 35 mbar of O_2 for 30 min. This treatment should result in the compensation of O vacancies in the surface and subsurface regions. So the metallic peak must not be observed if the vacancy free surface was prepared. If the UV irradiation reads to the surface O vacancies and a metallic band appears, it is possible to interpret that the 2DEG gives the band. The photon energy used in the present study was 128 eV, which is a very surface sensitive condition. Therefore, the authors must guarantee that the observed metallic band is the bulk CB.

No, there is no possibility that the band we observe arises from the 2DEG. Some of us have shown in a separate detailed ARPES study (S. Moser et

al., PRL 110, 19640 (2013)) that, unlike *e.g.* the case of $SrTiO_3$, oxygen vacancies in anatase TiO_2 actually do not yield a 2D surface electron gas, but rather introduce carriers into the conduction band. This can be clearly determined by the closed shape of the electron pocket along the k_z direction. In every measurement we performed we checked that the observed metallic band coincided with the bulk conduction band.

(5) How was the excess electron density determined? Transport measurements or ARPES?

The excess electron density is determined by both transport measurements and ARPES. We added this information in the revised version.

(6) "ii) a reduction of the oscillator strength of all peaks, ..." (p. 8) The peaks II and III are well above the Fermi level. Therefore, the reduction cannot be ascribed to the spectral weight transfer.

We respectfully disagree with this comment.
Firstly we note that the ε_2 spectra reported in the current Fig. 4 are trivially related to the σ_1 spectra, $\varepsilon_2 = 4\pi/\omega * \sigma_1$. A well-established sum-rule is that the total spectral weight (SW), defined as the integral of σ_1 between 0 and $+\infty$, is a constant due to the density of electrons in a material being a constant. Doping the pristine anatase TiO_2 single crystal eventually leads to redistribution of SW. For a standard insulator without strong electronic correlation (as for anatase TiO_2), this redistribution of SW is expected to

occur only towards lower energies. Different is the case of a strongly correlated electron system, where the SW can also be redistributed to high energies of the order of the Mott-Hubbard U. In our case, doping the material with n = 2 x 10^{19} cm^{-3} via oxygen vacancies leads to: i) the presence of a higher density of oxygen vacancies; ii) the shift of the Fermi energy inside the conduction band, close to the bottom at the Γ point.

Effect i) gives rise to the tail below the fundamental gap of the material, because of the increased probability of defect-assisted interband transitions. Effect ii) leads to an increased density of electrons in the conduction band, which occupy states in a wide portion of the Brillouin zone around Γ. Peaks II and III arise from resonant or excitonic (interband) transitions involving occupied states in the valence band and unoccupied states in the conduction band. While the occupied states involved in these transitions belong to deeper valence bands, the unoccupied states still involve the Γ-Z direction. The increased density of electrons in the conduction band produces a band filling mechanism (i.e. fewer available unoccupied states for the interband transition), which in turn reduces the SW of peaks II and III.

The increased doping is also expected to produce a larger Drude contribution from free carrier absorption, and this takes up the lost SW from the high-energy region. This is the standard behaviour in most semiconductors and insulators and anatase TiO_2 is not showing any anomalous behaviour in this regard.

(7) "The insensitivity of peak I for different doping levels" (p. 11) "The insensitivity" of what? Energy or intensity?

We thank the referee for this comment, as the sentence was not complete. We modified it specifying that the "insensitivity" refers to the energy.

(8) "As far as peak I is concerned, it extends two-dimensionally on the (001) plane with a radius of 15 angstrom, being almost confined to a single atomic plane along the c-axis." (p. 12) From Fig. 3a, the radius of the exciton distribution is much larger than 1.5 nm. From Fig. 3d, the distribution seems not to be confined in a single plane but in two planes. Not "along the c-axis", but "in the ab-plane".

In the revised version of the paper, we clarified the meaning of the spatial parameters that we provide. The radius of 1.5 nm reported in the previous version of the manuscript actually corresponds to the point at which 90% of the square absolute value of the exciton wavefunction is contained and the figure is fully compatible with this value. In the text, now we also report the Bohr radius of the exciton wavefunction (considering a 2D hydrogen model), since this is a well-defined and unambiguous quantity. We obtain an average value of 3.2 nm for the Bohr radius.

(9) The binding energy of exciton III is evaluated to be 150 meV (p. 13). How was this value deduced?

The binding energy of exciton III was evaluated from the onset of the continuum in the RPA @ GW spectra, further corrected for the presence of the electron-phonon interaction. We specified this more clearly in the revised version of the paper.

(10) Since Fig. 4b shows absorption spectra of only one doped (or pristine) TiO_2 sample, "anatase TiO_2 single crystal (with varying degrees of oxygen vacancies)" (p. 15) is misleading.

We agree that the sentence might have generated some confusion. The data displayed in Fig. 4b have indeed been obtained for the doped (n = 2 x 10^{19} cm^{-3}) sample. The sentence refers to the generality of these findings for all the samples studied via transient reflectivity (see the similarity of the transient reflectivity signals in Fig. S7 of the revised SI). Thus, despite the fact that the sentence in the previous version of the manuscript is correct, we decided to rephrase it to avoid confusion.

(11) "Both features have a relatively narrow width of 250 meV." (p. 15) I see the peak is much broader than 0.25 eV (Fig. 4).

In the revised version of the paper we made a more accurate estimate of the linewidth by fitting the excitonic features of Fig. 4 with a Lorentzian shape. The new values are reported in the text.

(12) "The reduced (pristine) form of anatase TiO_2" (p. 18) may lead confusion. "Pristine crystal" and "n-doped crystal" should be clearly defined with the excess electron densities. The density of the Cu-doped crystal is also desired.

We clarified all these issues by specifying the doping level determined for all the 3 types of single-crystals used in our study.

(13) The photon energy used for the ARPES measurements must be indicated in the main text, though it is offered in the supplemental. The energy resolution of the system is also desired.

We added the required information in the main text.

(14) Typos: p. 10; (Fig. 2a-b, red line) → (Fig. 2c-d, red line) p. 20; 5% fluoridic acid → 5% fluoric acid or 5% hydrofluoric acid

We thank the referee for her/his very careful and critical reading of our manuscript. We also fixed these typos.

REVIEWERS' COMMENTS:

Reviewer #4 (Remarks to the Author):

As I wrote earlier, the present manuscript clearly improves on previous experimental and theoretical characterizations of the electronic structure and optical properties anatase TiO_2. In the moment it certainly represents the most authoritative description available. Given the widespread use of this material in a broad range of applications, it will certainly attract a wide readership. My major concern with the previous version of this manuscript was that it was lacking substantial new physical insight, but rather was a mere incremental improvement over previous work. The authors made an effort to address this issue, deepened the physics discussion and now provide arguments that may explain the appearance of excitons that are spatially confined to two dimensions in a three-dimensional material. The discussion on this point is not really compelling. Still, the manuscript has improved and I view the paper now as borderline acceptable for Nature Communications.

Reviewer #5 (Remarks to the Author):

The authors' response to my questions and comments is satisfactory, and the manuscript is properly revised. Especially, enrichment of the discussion about physics of the exciton makes the manuscript significantly valuable. Moreover, my main concern about the novelty of the paper is resolved by largely softening the claim of the 2D distribution of the exciton. I believe that the manuscript contributes to deepen our understanding of optical responses of wide band gap materials. Therefore, the revised version seems to meet the criteria of Nat. Commun.

There are two minor comments.

(1) The energy scale in Figs. 3 and 5 is referenced to the bottom of the conduction band. On the other hand, the Fermi level is zero in the energy scale of Fig. 1 and Supplementary Fig. 3. I recommend the authors to clearly distinguish these two energy scales by, for example, changing the title of the axis.

(2) It is better to avoid using angstrom because nm is more frequent in the present manuscript.

REVIEWERS' COMMENTS:

Reviewer #4 (Remarks to the Author):

As I wrote earlier, the present manuscript clearly improves on previous experimental and theoretical characterizations of the electronic structure and optical properties anatase TiO2. In the moment it certainly represents the most authoritative description available. Given the widespread use of this material in a broad range of applications, it will certainly attract a wide readership.

We thank the referee for this comment.

My major concern with the previous version of this manuscript was that it was lacking substantial new physical insight, but rather was a mere incremental improvement over previous work. The authors made an effort to address this issue, deepened the physics discussion and now provide arguments that may explain the appearance of excitons that are spatially confined to two dimensions in a three-dimensional material. The discussion on this point is not really compelling. Still, the manuscript has improved and I view the paper now as borderline acceptable for Nature Communications.

In our revised manuscript we made an effort to understand the origin of strongly bound excitons in such a wide bandgap insulator. Concerning the novelty of our work in its previous forms, the referee should consider also

the inclusion of the ultrafast data on the nanoparticles. We believe this was not an incremental improvement over previous works but an important achievement by itself. Indeed, no clear absorption peaks have ever been resolved in the steady-state absorption spectra of these nanomaterials. This result has been possible only through the use of state-of-the-art experimental technique, such as ultrafast spectroscopy in the deep-UV range. Concerning the discussion provided about the physics of the excitons, we believe that our conclusions go well beyond the established theory of excitons provided by Toyozawa. We are not aware of any paper in the literature reporting an intuitive but rigorous explanation for the discrepancies in the exciton binding energies of wide-bandgap materials. The most advanced explanation is the one provided in M. Dvorak et al., Phys. Rev. Lett. 110, 016402 (2013). However, the cases of anatase TiO_2 and $SrTiO_3$ do not follow the curve reported in Fig. 1. Just a proper consideration of the electronic band structure and (especially) the screening effects with respect to the binding energy can rationalize the general scenario.

Reviewer #5 (Remarks to the Author):

The authors' response to my questions and comments is satisfactory, and the manuscript is properly revised. Especially, enrichment of the discussion about physics of the exciton makes the manuscript significantly valuable. Moreover, my main concern about the novelty of the paper is resolved by largely softening the claim of the 2D distribution of the exciton. I believe that the manuscript contributes to deepen our

understanding of optical responses of wide band gap materials. Therefore, the revised version seems to meet the criteria of Nat. Commun.

We thank the referee for her/his appreciation of our work.

There are two minor comments.

(1) The energy scale in Figs. 3 and 5 is referenced to the bottom of the conduction band. On the other hand, the Fermi level is zero in the energy scale of Fig. 1 and Supplementary Fig. 3. I recommend the authors to clearly distinguish these two energy scales by, for example, changing the title of the axis.

We modified the figures as requested by the referee, distinguishing the two energy scales by changing the title of the axis.

(2) It is better to avoid using angstrom because nm is more frequent in the present manuscript.

We followed the referee's suggestion and changed all the sizes of the exciton wavefunctions from angstrom to nm. Only the lattice constants were maintained in angstrom to be consistent with the international convention.
